# Lipocalin-2-mediated ferroptosis as a target for protection against light-induced photoreceptor degeneration

**DOI:** 10.1186/s10020-025-01250-1

**Published:** 2025-05-15

**Authors:** Wenyi Tang, Ruyi Zhai, Jun Ma, Gezhi Xu

**Affiliations:** 1 Eye Institute and Department of Ophthalmology, Eye & ENT Hospital, Fudan University, Shanghai, 200031 China; 2 Shanghai Key Laboratory of Visual Impairment and Restoration, Shanghai, 200031 China; 3 NHC Key Laboratory of Myopia and Related Eye Diseases; Key Laboratory of Myopia and Related Eye Diseases, Chinese Academy of Medical Sciences, Shanghai, 200031 China

**Keywords:** Lipocalin 2, Ferroptosis, Photoreceptor, Light-induced retinal degeneration, JNK

## Abstract

**Background:**

Retinal degeneration is a leading cause of blindness worldwide. The induction of ferroptosis has been identified as an important mechanism contributing to the loss of photoreceptors in retinal degeneration. Lipocalin-2 (LCN2) exhibits iron-regulatory properties and may modulate cell viability in various diseases. However, the effects of LCN2 on ferroptosis in retinal degeneration remain unclear.

**Methods:**

A light-induced injury model using 661W photoreceptor cells and a light-induced retinal degeneration male rat model were established. LCN2 protein expression was assessed by western blotting. The effects of LCN2 on ferroptosis in vitro were investigated by using recombinant LCN2 protein (rLCN2) and small-interfering RNA (siRNA) targeting LCN2 (siLCN2). Fe^2+^, malondialdehyde (MDA), tripeptide glutathione (GSH) levels, and the expression of ferroptosis-associated proteins (solute carrier family 7 member 11 [SLC7A11] and glutathione peroxidase-4 [GPX4]) were measured. A phosphokinase array and western blotting were performed to elucidate the mechanisms underlying LCN2-modulated photoreceptor ferroptosis. Additionally, the protective effects of LCN2 knockdown using adeno-associated virus (AAV)-expressing short hairpin RNA (shRNA) targeting LCN2 (AAV-shRNA-LCN2) on retinal structure and function in vivo were evaluated by hematoxylin and eosin staining and electroretinography.

**Results:**

LCN2 expression was significantly upregulated following light exposure. Treatment with rLCN2 significantly induced ferroptosis in photoreceptor cells, as shown by decreased cell viability, increased Fe^2+^ levels, inhibition of SLC7A11 and GPX4 expression, depletion of GSH, and enhanced MDA levels, whereas siLCN2 protected against these effects. Exposure of photoreceptor cells to rLCN2 activated c-Jun N-terminal kinase (JNK), and administration of the JNK inhibitor SP600125 protected photoreceptor cells from ferroptosis. Lastly, AAV-shRNA-LCN2 administration inhibited light-induced ferroptosis in the retina, and protected the retinal structure and function in vivo.

**Conclusion:**

LCN2 is a key regulator of light-induced ferroptosis in photoreceptors by modulating the JNK pathway. Therefore, LCN2 presents a new target for the treatment of retinal degeneration.

**Supplementary Information:**

The online version contains supplementary material available at 10.1186/s10020-025-01250-1.

## Introduction

The primary and secondary death of photoreceptors that occurs in retinal degenerative diseases, including retinitis pigmentosa (RP) and age-related macular degeneration (AMD), results in major visual impairment and blindness (Curcio et al. 1996; Nair and Thomas 2022). Protecting the photoreceptors is critical for preserving vision in these diseases. However, the underlying molecular mechanisms remain inadequately understood (Zhang et al. 2024). Excessive light exposure-induced photoreceptor degeneration serves as an established experimental model for studying human atrophic AMD and RP (Marc et al. 2008; Reme et al. 1998). In this model, prolonged actinic light triggers photoreceptor death through multiple mechanisms including rhodopsin photobleaching, dysregulated retinoid processing, and excessive generation of reactive oxygen species (ROS)—processes that partially recapitulate key features of human retinal degeneration (Marc et al. 2008). While apoptosis has been well-documented in both light-induced and hereditary retinal degeneration (Chang et al. 1993; Gu et al. 2017; Ni et al. 2008), accumulating evidence suggests the involvement of additional cell death mechanisms. Perche et al. demonstrated that caspase inhibition using Z-VAD (targeting caspases including caspase-1 and caspase-3) only partially attenuated light-induced photoreceptor degeneration (Perche et al. 2007). Similarly, Yoshizawa et al. found that systemic administration of the caspase-3 inhibitor Ac-DEVD-CHO provided merely transient and modest protection in C3H mice with inherited retinal degeneration (Yoshizawa et al. 2002). These observations strongly suggest the contribution of non-apoptotic cell death pathways in photoreceptor degeneration (Murakami et al. 2013), highlighting the need to investigate alternative mechanisms in retinal pathology.

Ferroptosis, a process distinct from necrosis, apoptosis, and autophagic cell death, is a newly identified type of programmed cell death that is characterized by iron accumulation and subsequent lipid peroxidation (Dixon et al. 2012). With the accumulation of labile ferrous ion (Fe^2+^) and inactivation of endogenous antioxidant system including glutathione peroxidase 4 (GPX4), ferroptosis can be triggered, leading to the accumulation of toxic lipid ROS and lipid peroxidation products such as malondialdehyde (MDA) (Dixon et al. 2012; Magtanong et al. 2016; Wang et al. 2023; Zhang et al. 2018). Photoreceptors, the primary sensory neurons involved in vision, are particularly vulnerable to lipid peroxidation due to their high energy demands and the abundance of polyunsaturated fatty acids (Brito et al. 2024). Ferroptosis has been implicated in photoreceptor degeneration in multiple retinal disease models, including AMD (Chen et al. 2021), RP (Xiong et al. 2023), retinal ischemia–reperfusion injury (Wang et al. 2023), diabetic retinopathy (Gao et al. 2023; Liu et al. 2024), and retinal detachment (Ye et al. 2025). Chen et al. demonstrated that all-*trans*-retinal (atRAL)-induced photoreceptor degeneration involved ferroptotic mechanisms mediated by increased Fe^2+^ accumulation, upregulation of long-chain acyl-coenzyme A synthase 4, inhibition of the cystine/glutamate antiporter (system Xc⁻), and mitochondrial dysfunction (Chen et al. 2021). Their subsequent mechanistic investigation revealed that heme oxygenase-1 activation exacerbated ferroptosis by elevating intracellular Fe^2+^ levels, which subsequently drove Fenton reaction-mediated ROS generation and lipid peroxidation (Chen et al. 2023). Our previous studies revealed that ferroptosis contributes to photoreceptor loss in light-induced retinal degeneration, and that ferrostatin-1, an inhibitor of ferroptosis, protected the retinal structure and function (Tang et al. 2021a). However, the mechanisms underlying the induction of ferroptosis in photoreceptors remain unclear.

The protein lipocalin-2 (LCN2), also known as neutrophil gelatinase-associated lipocalin (NGAL) or 24p3, is a member of the adipokine family of proteins (Flower 1996). LCN2 can bind to and transport small molecules, such as retinoids, fatty acids, steroids, and iron (Xiao et al. 2017). As a pleiotropic regulator of iron metabolism and oxidative stress (Xiao et al. 2017), LCN2 has been increasingly recognized as a critical mediator of ferroptosis. Mechanistically, LCN2 exacerbates ferroptotic cell death by facilitating iron-dependent lipid peroxidation. In neurons, LCN2 enhanced intracellular Fe^2+^ accumulation, thereby amplifying oxidative damage and ferroptosis (Wang et al. 2024). Similarly, in cardiomyocytes, LCN2 elevated ROS levels and potentiated erastin-induced ferroptosis (Jiang et al. 2025). Furthermore, Liu et al. demonstrated that LCN2 suppressed the system Xc⁻, leading to diminished cystine uptake and GSH depletion, which collectively compromised cellular antioxidant defenses (Liu et al. 2023b). Research on the retina suggested that LCN2 contributed to retinal ganglion cell ferroptosis in ischemic retinopathy, although the precise molecular mechanisms remain to be fully elucidated (Mei et al. 2023). Additionally, a recent study revealed that increased LCN2 in the retinal pigment epithelium (RPE) inhibited autophagy and deregulated iron homeostasis, resulting in inflammasome activation, oxidative stress, and ferroptosis in RPE cells (Gupta et al. 2023). However, the role of LCN2 in ferroptosis in photoreceptor cells in light-induced retinal degeneration has not been explored.

The c-Jun N-terminal kinase (JNK) pathway has been implicated in ferroptosis regulation in multiple pathological contexts (Varga et al. 2022). For instance, in protein kinase D-mediated cardiac hypertrophy, JNK/p53 signaling drove ferroptosis by suppressing the solute carrier family 7 member 7 (SLC7A11)/GPX4 axis (Lv et al. 2024). Similarly, in atRAL-induced retinal degeneration, JNK activation exacerbated photoreceptor ferroptosis via NCOA4-dependent ferritinophagy (Yang et al. 2025), underscoring the pathway’s direct role in ferroptotic cell death. Notably, LCN2 has been shown to modulate JNK signaling in diseases such as cancer (Huang et al. 2024; Lee et al. 2011) and acute liver injury (Borkham-Kamphorst et al. 2013). However, whether LCN2 regulates JNK activity in retinal degeneration, and the mechanistic link between JNK activation and light-induced photoreceptor ferroptosis, remain unresolved.

This study elucidates the role of LCN2 in mediating ferroptosis during light-induced photoreceptor degeneration through complementary in vitro and in vivo approaches. For in vitro experiments, we employed the well-characterized 661 W immortalized cone photoreceptor cell line, which has been extensively validated for modeling light-induced damage, photo-oxidative stress responses, and ferroptosis pathways (Tang et al. 2021a, b; Tsuruma et al. 2012). Parallel in vivo studies were conducted using adult albino Sprague–Dawley rats, an established model system exhibiting well-documented susceptibility to blue light-induced retinal damage (Zhang et al. 2012; Tang et al. 2021a, b; Tang et al. 2018b). Furthermore, we investigated the mechanism through which LCN2 induces ferroptosis, and specifically identified its association with iron overload and GPX4 depletion resulting from activation of the JNK signaling pathway.

## Material and methods

### Cell culture and treatment

The murine photoreceptor cell line 661 W was obtained from Jennio Biotech Co., Ltd. (Guangzhou, China) and cultured in Dulbecco’s modified Eagle’s medium (DMEM; Gibco, Carlsbad, CA, USA) containing 4.5 g/L of glucose and 10% fetal bovine serum (Thermo Fisher Scientific, Waltham, MA, USA) at 37 °C in a 5% CO_2_ atmosphere. The cells were inoculated in six- or 24-well plates, and the culture medium was replaced with serum-free medium once the cell density reached 90%. For light exposure, the cells were incubated at a constant temperature of 37 °C and exposed to blue light (6000 lx) for 5 h as previously reported (Zhang et al. 2012). Control cells were placed in a dark box within the same incubator. After light exposure, the cells were returned to the standard incubator for subsequent experiments. For drug treatment, recombinant LCN2 protein (rLCN2; R&D Systems, Minneapolis, MN, USA) and the JNK inhibitor SP600125 (Abmole, Houston, TX, USA) were dissolved in phosphate-buffered saline (PBS) and dimethyl sulfoxide (DMSO; Sigma-Aldrich, St. Louis, MO, USA), respectively, and were administered to 661 W cells for 24 h at the indicated concentrations. The final concentration of DMSO was < 0.1%. For rLCN2 and SP600125 cotreatment, SP600125 (5 µM) was added 30 min before the addition of rLCN2 (1 µg/mL).

### Cell viability

Cell viability was assessed using a Cell Counting Kit-8 (CCK-8) (Dojindo, Kumamoto, Japan). 661 W cells were plated in 96-well culture plates at a density of 5 × 10^3^ cells/well. Following light exposure or rLCN2 treatment at the indicated times, the culture medium was replaced and 10% CCK-8 solution was added. The cells were then incubated at 37 °C for 1 h and absorbance was measured at 450 nm using a microplate reader (TECAN, Zurich, Switzerland).

### Western blotting

Total protein was extracted from each sample using RIPA lysis buffer (Beyotime, Shanghai, China) supplemented with protease and phosphatase inhibitors (Beyotime). Equal amounts of protein (30 μg) from different groups were separated by gel electrophoresis using 15% sodium dodecyl sulfate–polyacrylamide gel electrophoresis gels (Tanon, Shanghai, China) and transferred to polyvinylidene fluoride membranes (Millipore, Billerica, CA, USA). The membranes were blocked with 5% non-fat milk for 1 h at 37 °C, and incubated overnight at 4 °C with the following primary antibodies: LCN2 (1:1000, ab216462, Abcam, Cambridge, UK), GPX4 (1:1000, ab125066, Abcam), SLC7A11 (1:1000, ab307601, Abcam), phosphorylated JNK (1:1000, 4668, Cell Signaling Technology, Danvers, MA, USA), JNK (1:1000, 9252, Cell Signaling Technology), and β-actin (1:1000, 4970S, Cell Signaling Technology). The membranes were then probed with appropriate horseradish peroxidase-conjugated secondary antibodies at room temperature for 1 h, followed by development using enhanced chemiluminescence (Millipore). Images were captured using an automatic gel imaging analysis system (Peiqing Science and Technology Co., Ltd., Shanghai, China) and analyzed with ImageJ software (National Institutes of Health, Bethesda, MD, USA). Western blotting raw data were provided as Additional file 1.

### Transmission electron microscopy (TEM)

661 W photoreceptor cells, cultured in six-well plates, were incubated with either 1 µg/mL rLCN2 or vehicle (PBS) for 24 h. The cells were then digested using a 0.25% trypsin‒ethylenediaminetetraacetic acid solution (Gibco), collected in sterile 1.5 mL tubes, and fixed overnight at 4 °C using fixation solution for TEM (Servicebio Technology, Hubei, China). After pre-embedding with 1% agarose, the cells were post-fixed with 1% OsO_4_ for 2 h at room temperature. The samples were then dehydrated using a series of ethanol concentrations ranging from 30 to 100%. Following Epon 812 resin (Structure Probe, Inc., West Chester, PA, USA) penetration, embedding, and polymerization, the samples were sectioned and stained with 2% uranyl acetate and 2.6% lead citrate. Images were captured using a transmission electron microscope (HT-7800, Hitachi, Tokyo, Japan).

### Measurement of Fe^2+^ content

The Fe^2+^ content was measured using a colorimetric iron assay kit (ab83366, Abcam). Briefly, 661 W cells or neural retinal tissues were homogenized in cold iron assay buffer and subsequently centrifuged at 16,000 × *g* for 10 min at 4 °C. The supernatant was collected, and the protein levels were measured using a bicinchoninic acid (BCA) protein assay kit (Beyotime). The samples were then incubated with 100 μL of iron probe for 60 min at 37 °C in the dark. The optical density at 593 nm was measured using a microplate reader (TECAN). The results were normalized to the protein concentration.

### Measurement of intracellular ROS

Intracellular ROS production was assessed using the 2′,7′-dichlorodihydrofluorescein diacetate (DCFH-DA) assay (Beyotime). In this assay, DCFH is oxidized to fluorescent dichlorofluorescein by ROS. 661 W cells were cultured in 24-well plates and treated as specified. Following treatment, the cells were rinsed with PBS and incubated with 5 μM DCFH-DA at 37 °C for 15 min in the dark. After washing the cells three times in PBS, 500 μL of Hoechst 33342 staining solution (Beyotime) was added to each well for 5 min at room temperature. The cells were then washed three times with PBS, and observed using a fluorescence microscope (DMi8, Leica Microsystems, Wetzlar, Germany). ROS levels were defined as the proportion of green cells and the data were analyzed using Image J software as previously reported (Xu et al. 2022).

### Measurement of MDA

Lipid peroxidation was assessed by quantifying the MDA concentration in cells or neural retinal lysates using an MDA assay kit (Beyotime). Following the indicated treatments, 661 W cells or retinal tissues were collected and lysed using RIPA lysis buffer (Beyotime) on ice. After centrifugation at 12,000 × *g*, the protein concentrations were determined using a BCA protein assay kit (Beyotime). The test solution was added to the supernatants and incubated at 100 °C for 15 min. The samples were centrifuged at 1000 × *g* and the supernatants were transferred to 96-well plates for measurement at 532 nm using a microplate reader (TECAN). The results were normalized to the total protein concentration.

### Measurement of GSH

The GSH concentrations in cell or neural retinal lysates were determined using a GSH assay kit (Beyotime) according to the manufacturer’s protocol. Briefly, the samples were lysed using protein removal reagent M solution and centrifuged at 10,000 × *g* at 4 °C for 10 min. The working solution was added to the supernatants and incubated at 25 °C for 5 min. The mixtures were then transferred to 96-well plates and the optical density was measured at 412 nm using a microplate reader (TECAN). The results were expressed as percentages of the control values.

### Preparation of small-interfering RNA

Small-interfering RNA (siRNA) targeting LCN2 (siLCN2) and non-targeting negative control (siNC) were prepared by Hanbio (Shanghai, China). 661 W cells were incubated overnight in a six-well plate at a density of 5 × 10^4^ cells/well. Following the manufacturer’s instructions for the RNAFit RNA transfection reagent (Hanbio), 10 μL of siRNA (either siLCN2 or siNC) was added to 200 μL of Opti-MEM and mixed gently three times. Subsequently, 30 μL of RNAFit was added to the mixture and vortexed for 10 s, followed by incubation for 10 min at room temperature to allow the siRNA and RNAFit to form a transfection complex. Concurrently, the medium was replaced with 1.8 mL of fresh DMEM supplemented with 10% FBS. The transfection complex was then added to the plate. The final volume of culture medium in each well was 2 mL, resulting in a final siRNA concentration of 50 nM. Subsequent experiments were performed 48 h post-transfection. The siRNA sequences were as follows: siNC, sense 5′-UUCUCCGAACGUGUCACGUTT-3′, antisense 5′-ACGUGACACGUUCGGAGAATT-3′; siLCN2, sense 5′-CGAUGUACAGCACCAUCUATT-3′, antisense 5′-UAGAUGGUGCUGUACAUCGTT-3′.

### Phosphokinase array

To investigate the potential mechanism underlying LCN2-mediated ferroptosis, we used phosphokinase arrays, in which the capture and control antibodies were spotted in duplicate onto nitrocellulose membranes. In preparation for the proteome profiling array experiment, 661 W cell lysates were obtained after treatment with LCN2 (1 μg/mL for 24 h) or PBS using lysis buffer supplemented with protease and phosphatase inhibitors. For each cell lysate, 500 μg of total protein was analyzed using a phosphokinase array kit (R&D Systems) according to the manufacturer’s instructions. The density of each spot was measured using ImageJ software and normalized against control signals on the same immunoblot membrane.

### Animals

All experiments were conducted in accordance with the Association for Research in Vision and Ophthalmology statement for the Use of Animals in Ophthalmic and Vision Research. The study was approved by the Animal Ethics Committee of the Eye and ENT Hospital of Fudan University. Six-week-old male Sprague–Dawley rats (Jiesijie Laboratory, Shanghai, China), weighing 160‒180 g, were housed at 25 ± 2 °C under regular lighting conditions (12-h light/dark cycle) with unrestricted access to standard diet and water. A total of 75 rats were used in the study and animal number in each group was listed in Additional file 2.

### Light-induced retinal degeneration model

After dilating the rats’ pupils with 1% atropine, the rats were dark-adapted for 12 h and exposed to 2500 lx of blue light for 24 h, as previously reported (Zhang et al. 2012). The rats were then returned to a normal 12-h light/dark cycle for 1, 3, and 7 days until subsequent analyses. During light exposure, the rats were provided free access to food and water, and the room temperature was maintained at 25 ± 1 °C. Control rats were dark-adapted for 24 h before being returned to the 12-h light/dark cycle. All rats were anesthetized by intraperitoneal injection of ketamine (200 mg/kg) and xylazine (10 mg/kg), and were then sacrificed for analysis. Considerable efforts were made to minimize the number of animals used and their discomfort throughout the study.

### LCN2 shRNA-AAV transduction

Adeno-associated virus (AAV) 2/2-EGFP containing short hairpin RNA for LCN2 (AAV-shLCN2) at a concentration of 1.3 × 10^12^ vg/mL and an AAV2/2-EGFP negative control containing scrambled short hairpin RNA (AAV-shNC) at a concentration of 1.4 × 10^12^ vg/mL were synthesized by Hanbio. Three short hairpin RNAs (shRNAs) targeting LCN2 were designed, and the shRNA with the best efficiency to knockdown LCN2 expression in the retina was selected. The sequences of AAV-shLCN2 and AAV-shNC are presented in Additional file 3. For subretinal injection under a surgical microscope, a sterile 30-gauge needle (Kindly Medical Devices, Zhejiang, China) was initially used to make a hole 1 mm posterior to the superior limbus. Subsequently, a 33-gauge needle (Hamilton, Reno, NV, USA) was used to inject AAV-shLCN2 or AAV-shNC subretinally through the hole. The needle tip was then positioned within the subretinal space, and approximately 3 μL of the viral suspension was injected to induce a bullous retinal detachment in the superior hemisphere. Topical ofloxacin eye ointment was then applied. After subretinal injection of AAV, the rats were maintained normally for three weeks to allow sufficient retinal transfection before transfection efficiency assessment or light exposure.

### Intravitreal administration of JNK inhibitor

SP600125 was prepared as a stock solution in DMSO and diluted with PBS to achieve a final working concentration of 40 μM, consistent with previously established protocols (Wu et al. 2020). For intravitreal administration, 5 μL of solution (yielding an estimated 0.2 nM concentration in the vitreous cavity) was injected using a sterile 33-gauge needle 1 mm posterior to the superior limbus. Following injection, animals were subjected to blue light exposure and subsequently sacrificed for analysis at indicated times.

### Isolation of rat neural retina

Following euthanasia, rat eyes were immediately enucleated and placed in ice-cold PBS. Using curved micro scissors, we performed a circumferential limbal incision to separate the anterior segment (including cornea, iris, and lens) from the posterior eyecup. After careful removal of vitreous humor, the posterior eyecup containing neural retina and RPE was gently rinsed with PBS. Under surgical microscope guidance, blunt-end forceps were inserted into the subretinal space to meticulously separate the neural retina from underlying RPE and sclera. The isolated neural retina was then immediately snap-frozen in liquid nitrogen and stored at −80 °C for subsequent molecular analyses.

### Immunofluorescence

The enucleated eyes were immersed in 4% paraformaldehyde, dehydrated by 20% and 30% sucrose solutions, and embedded in optimal cutting temperature compound (Tissue-Tek, Tokyo, Japan). Cryosections were sagittally cut through the optic disc. The sections were rinsed with PBS and treated with PBS plus 0.1% TritonX-100 for 15 min, which was followed by blocking with 5% goat serum for 1 h at room temperature. Primary antibody for LCN2 (1:100, ab216462, Abcam) was incubated at 4 °C overnight. After three washes with PBS, immunofluorescent secondary antibody (Alexa Fluor 555, 1:1000, A27039, Thermo Fisher Scientific) was incubated for 1 h at room temperature. DAPI (Beyotime) was applied to stain nuclei. Images of the retinas at the same position under the same conditions were taken using a fluorescence microscope (Leica Microsystems).

### Hematoxylin and eosin staining

Seven days after light exposure, the rats were sacrificed and their eyeballs were immediately removed and fixed overnight in 4% paraformaldehyde, followed by dehydration, paraffin embedding, and sectioning along the sagittal plane. Sections (3 μm thick) encompassing the optic papilla were stained with hematoxylin and eosin (H&E) and examined under a light microscope. The thickness of the outer nuclear layer (ONL) was measured and the number of rows of photoreceptor cell nuclei were counted in 16 retinal areas, spaced 500 μm apart, using CaseViewer 2.4 software (3DHISTECH, Budapest, Hungary). The data of ONL thickness and nuclei rows were presented average from each eye. Images of the retinal areas that were most susceptible to light-induced injury, 1000–1500 μm superior to the optic papilla, were captured and are presented as representative samples, as previously reported (Kong et al. 2024).

### Electroretinography

Electroretinography (ERG) was performed 7 days after light exposure. The rats were subjected to 12-h dark adaptation, and then anesthetized with an intraperitoneal injection of ketamine (200 mg/kg) and xylazine (10 mg/kg). Full-field ERG was performed using a visual electrophysiology system (Espion E3, Diagnosys, Cambridge, UK) as previously described (Gu et al. 2017). Scotopic stimuli at 0.01, 0.1, 1.0, 3.0 and 10.0 cd·s/m^2^ and photopic stimuli at 10.0 cd·s/m^2^ were recorded. The amplitudes of the a and b waves were quantified by measuring from the prestimulus baseline to the nadir of the a wave and from the nadir of the a wave to the peak of the b wave, respectively.

### Statistical analysis

Statistical analyses were performed using GraphPad Prism 9.0.0 software (GraphPad, Boston, MA, USA) and IBM SPSS Statistics 29.0 (IBM, Armonk, NY). All results are presented as means ± standard deviation from a minimum of three independent experiments. The data were first assessed for normality using the Shapiro–Wilk test and for variance homogeneity using Levene's test. When the data were normally distributed (*P* > 0.05) and displayed homogeneity of variance (*P* > 0.1), Student’s *t*-test for comparisons between two groups or one-way analysis of variance (ANOVA) followed by Tukey’s post hoc test for multiple comparisons was used, as appropriate. Otherwise, nonparametric tests such as Mann–Whitney *U* (two-group comparisons) test or Kruskal–Wallis analysis with Dunn’s post hoc correction (multiple comparisons) were used. Statistical significance was defined as a *P*-value of < 0.05.

## Results

### Light exposure induced LCN2 and ferroptosis-related protein expression in 661 W cells

**Fig. 1:**
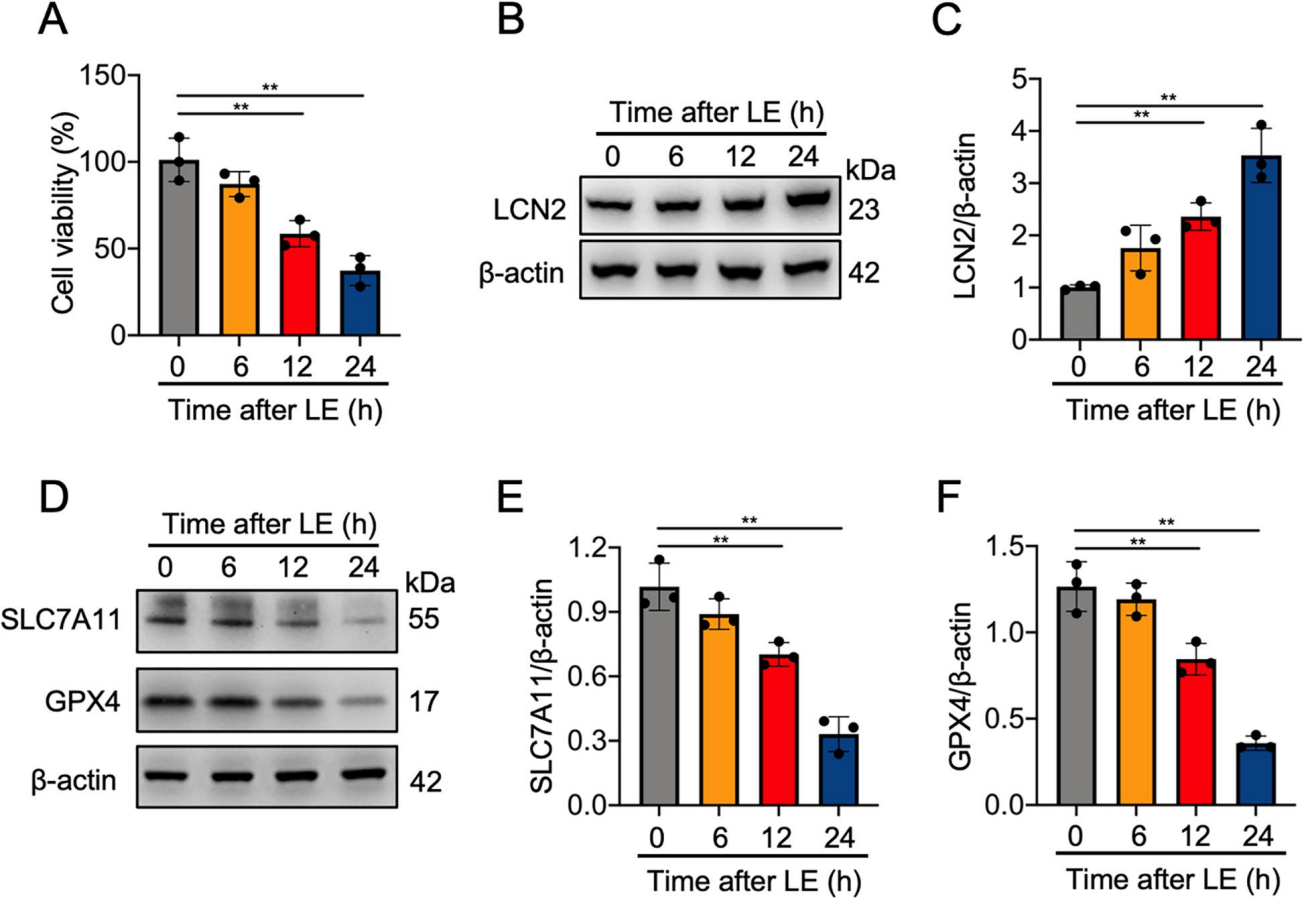
Light exposure (LE) induced the expression of LCN2 and ferroptosis-related proteins in 661 W photoreceptor cells. **A** Cell viability, at 6, 12, and 24 h after LE (6000 lx for 5 h). **B**, **C** Western blotting and quantitative analysis of LCN2 protein expression at 6, 12, and 24 h after LE. The protein expression levels of LCN2 were normalized to those of β-actin and are presented as fold changes. **D**‒**F** Western blotting and quantitative analysis of the protein expression of SLC7A11 and GPX4 at 6, 12, and 24 h after LE. The protein expression levels of SLC7A11 and GPX4 were normalized to those of β-actin and are presented as fold changes. *n* = 3 per group. *P < 0.05, **P < 0.01. One-way ANOVA followed by Tukey’s post hoc test

Exposure to light resulted in a gradual decline in the viability of 661 W cells, with significant decreases at 12 and 24 h after light exposure (Fig. 1A). Western blotting demonstrated that light exposure significantly increased LCN2 protein expression at 12 and 24 h compared with the control conditions (Fig. 1B,C). Evaluation of ferroptosis-related biomarkers revealed concomitant time-dependent decreases in both GPX4 and SLC7A11 protein expression, with statistically significant reductions observed at 12 h and 24 h after light exposure (Fig. 1D-F). These findings suggest that LCN2 may play a pathogenic role in light-induced ferroptosis in 661 W cells.

### LCN2 promoted ROS generation and triggered ferroptosis in 661 W cells

**Fig. 2:**
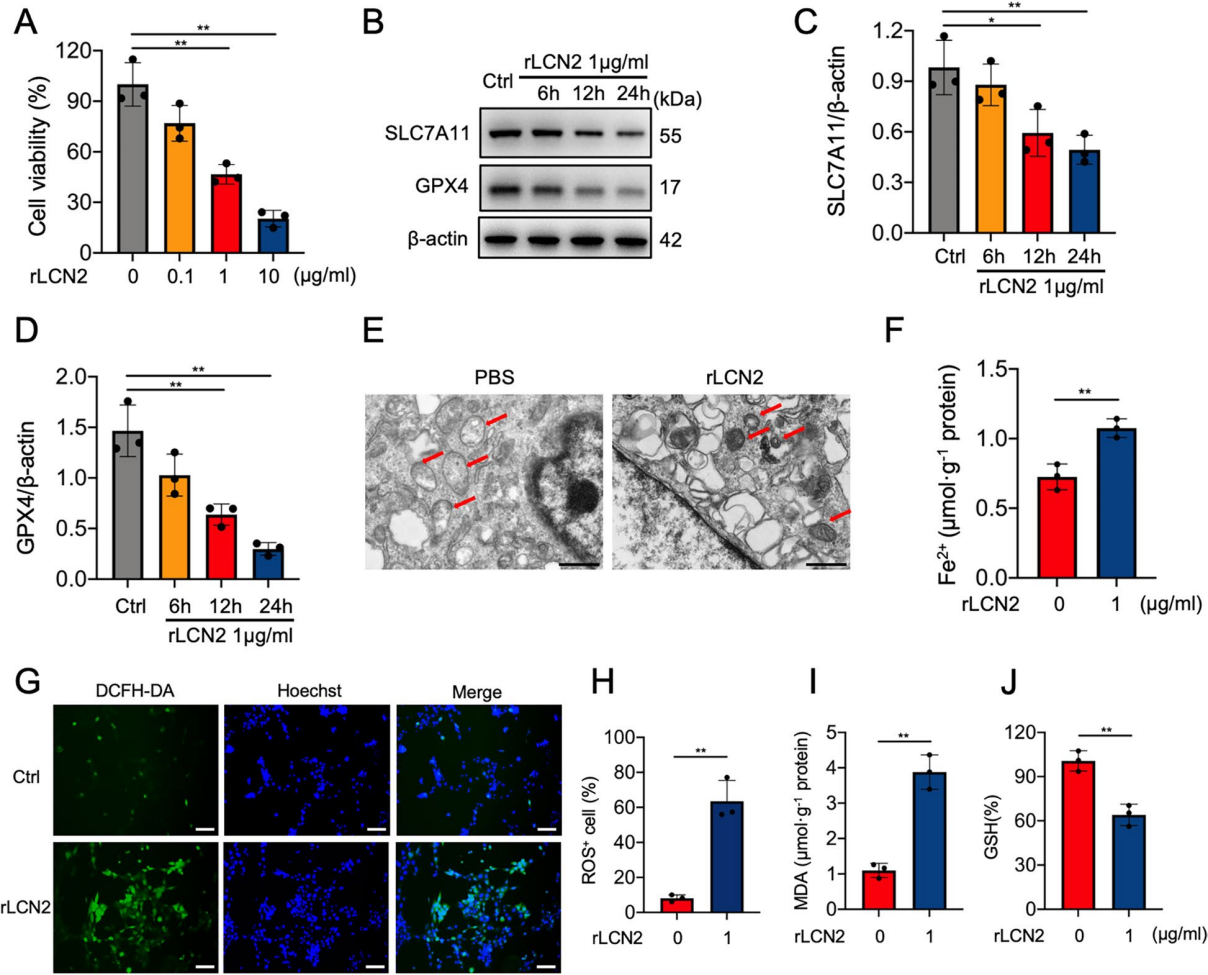
rLCN2 induced ferroptosis in 661 W photoreceptor cells. **A** Cell viability of 661 W cells incubated with serial concentrations of rLCN2 (0, 0.1, 1, and 10 μg/mL) for 24 h. **B**‒**D** Western blotting and quantitative analysis of SLC7A11 and GPX4 protein expression levels in 661 W cells exposed to 1 μg/mL rLCN2 for 24 h. The protein expression levels of SLC7A11 and GPX4 were normalized to those of β-actin and are presented as fold changes. **E** TEM images of mitochondria (arrows) in 661 W cells treated with 1 μg/mL rLCN2 for 24 h. Scale bars = 1 μm. **F** Intracellular Fe^2+^ levels, measured using a colorimetric iron assay kit, in 661 W cells treated with 1 μg/mL rLCN2 for 24 h. **G** Intracellular ROS levels, measured using DCFH-DA (green), in 661 W cells treated with 1 μg/mL rLCN2 for 24 h. Nuclei were stained blue with Hoechst 33342 dye solution. Scale bars = 50 μm. **H** Quantification of ROS levels as the proportion of green cells (%). **I** MDA levels in 661 W cells incubated with 1 μg/mL rLCN2 for 24 h. **J** GSH levels in 661 W cells after incubation with 1 μg/mL rLCN2 for 24 h. GSH levels are shown as a percentage of the levels in control cells. Cells treated with vehicle (PBS) alone served as the control group. *n* = 3 per group. *P < 0.05, **P < 0.01. One-way ANOVA followed by Tukey’s post hoc test for A-D, Student's *t*‐test for F, H-J

To investigate the potential effects of LCN2 on ferroptosis, 661 W cells were stimulated with rLCN2. The CCK-8 assay revealed that treatment with rLCN2 at concentrations of 1 and 10 μg/mL for 24 h significantly reduced the viability of 661 W cells (Fig. 2A). Western blotting revealed a gradual, time-dependent decline in the expression of SLC7A11 and GPX4 in cells treated with 1 μg/mL rLCN2, with significant reductions at 12 and 24 h (Fig. 2B-D). Considering these findings, we treated the 661 W cells with 1 μg/mL rLCN2 in subsequent experiments. TEM revealed that rLCN2 induced notable morphological changes, including shrunken mitochondria and condensed mitochondrial membrane (Fig. 2E), which are characteristic features of ferroptosis. The labile Fe^2+^ is the primary mediator of Fenton reactions and indicator of ferroptosis (Garcia-Baez et al. 2025; Jiang et al. 2021), so Fe^2+^ was measured in this study. Noticeably, rLCN2 induced an increase in the intracellular Fe^2+^ levels as measured by an iron assay kit (Fig. 2F). Consistently, rLCN2 treatment led to enhanced ROS production (Fig. 2G,H) and a significant elevation in MDA levels (Fig. 2I), a byproduct of lipid peroxidation. GSH is essential to physiological defenses against oxidative stress, and GSH levels were significantly reduced in 661 W cells exposed to 1 μg/mL rLCN2 for 24 h (Fig. 2J). These findings suggest that LCN2 may stimulate ferroptosis in photoreceptor cells.

### LCN2 knockdown alleviated light-induced ferroptosis in vitro

**Fig. 3:**
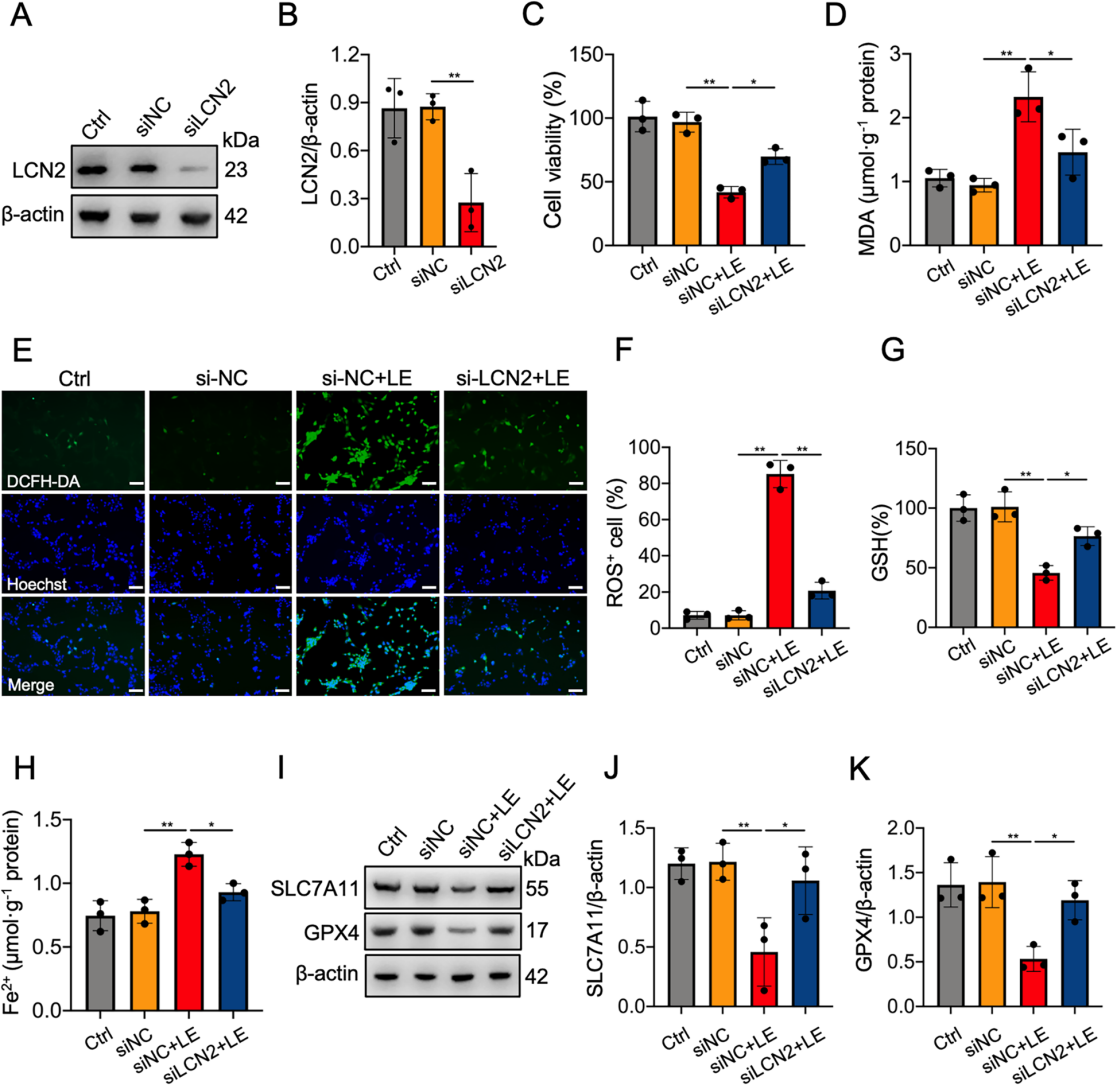
LCN2 knockdown alleviated light exposure (LE)-induced ferroptosis in 661 W photoreceptor cells. **A**, **B** Transfection of LCN2 siRNA confirmed by western blotting and quantitative analysis. The protein expression levels of LCN2 were normalized to those of β-actin and are presented as fold changes. **C** Cell viability of the indicated groups at 24 h after LE. **D** MDA levels of the indicated groups at 24 h after LE. **E** Intracellular ROS levels, measured using DCFH-DA (green), of the indicated groups at 24 h after LE. Nuclei were stained blue with Hoechst 33342 dye solution. Scale bars = 50 μm. **F** Quantification of ROS levels as the proportion of green cells (%). **G** GSH levels of the indicated groups at 24 h after LE. **H** Intracellular Fe^2+^ levels in the indicated groups at 24 h after LE. **I**‒**K** Western blotting and quantitative analysis of SLC7A11 and GPX4 protein expression in the indicated groups at 24 h after LE. The protein levels of SLC7A11 and GPX4 were normalized to those of β-actin and are presented as fold changes. *n* = 3 per group. *P < 0.05, **P < 0.01. One-way ANOVA followed by Tukey’s post hoc test

To provide further evidence that the activation of LCN2 by light exposure contributes to ferroptosis in photoreceptor cells, we knocked down LCN2 using siRNA in 661 W cells. Western blotting demonstrated that siLCN2 significantly reduced LCN2 protein expression compared with siNC (Fig. 3A,B). siLCN2 treatment increased the viability of light-exposed 661 W cells from 41.8% to 69.8% (Fig. 3C). Furthermore, siLCN2 significantly decreased ROS production and lipid peroxidation induced by light exposure (Fig. 3D-F). siLCN2 significantly reversed the reduction in GSH levels and the elevation in Fe^2+^ levels caused by light exposure (Fig. 3G,H). Moreover, western blotting revealed that the protein expression levels of SLC7A11 and GPX4 were decreased in light-treated cells but were upregulated following siLCN2 transfection (Fig. 3I-K). These results suggest that LCN2 knockdown protects 661 W cells from light-induced ferroptosis.

### LCN2 promoted ferroptosis in 661 W cells by activating JNK pathway

**Fig. 4:**
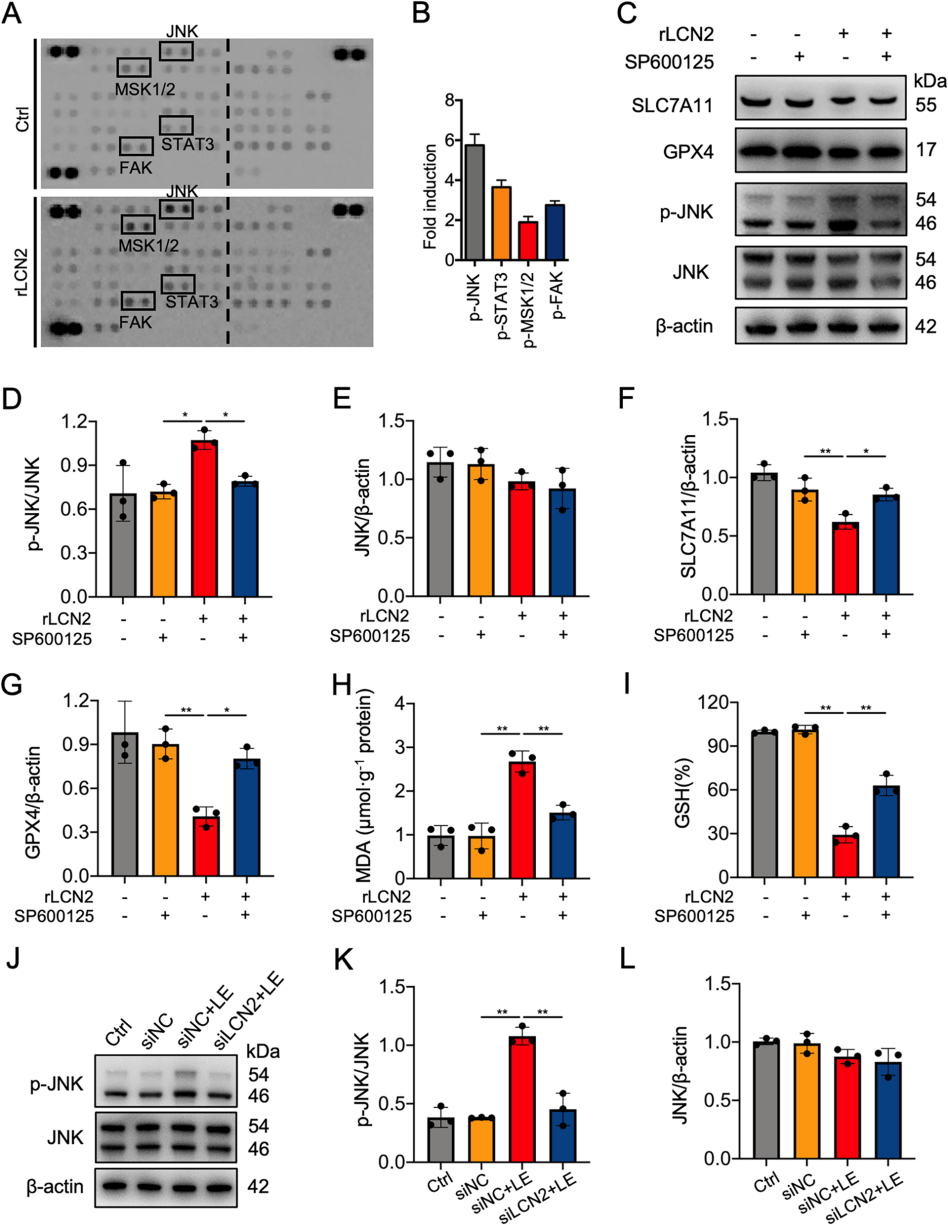
LCN2 regulated ferroptosis in 661 W photoreceptor cells by modulating the JNK pathway. **A, B** Phosphokinase array, revealing that treatment with 1 μg/mL rLCN2 for 24 h activated p-JNK expression compared with the vehicle control (Ctrl; PBS), which was validated by western blotting analysis. **C**‒**G **Western blotting and quantitative analysis of SLC7A11 and GPX4 protein expression levels in the indicated groups. 661 W cells were preincubated with SP600125 (5 μM) or vehicle (DMSO) for 0.5 h, followed by treatment with or without 1 μg/mL rLCN2 for 24 h. Protein expression levels of p-JNK were normalized to those of JNK and are presented as fold changes. The protein expression levels of JNK, SLC7A11, and GPX4 were normalized to those of β-actin and are presented as fold changes. **H **MDA levels in the indicated groups. **I **GSH levels in the indicated groups at 24 h after light exposure (LE). **J**‒**L **Western blotting and quantitative analysis, showing that LCN2 knockdown inhibited LE-induced JNK phosphorylation. Protein levels of p-JNK and JNK were normalized to those of JNK and β-actin, respectively, and are presented as fold changes.*n*= 1 for the phosphokinase array.*n*= 3 for western blotting, MDA, and GSH measurements per group. *P< 0.05, **P< 0.01. One-way ANOVA followed by Tukey’s post hoc test

Emerging evidence indicates that the JNK signaling pathway plays a regulatory role in ferroptosis and can be modulated by LCN2 (Huang et al. 2024; Lv et al. 2024). To investigate the mechanistic link between LCN2's pro-ferroptotic effects and JNK activation, we performed phosphokinase array analysis in 661 W photoreceptor cells. Our results demonstrated that treatment with rLCN2 notably induced JNK phosphorylation (Fig. 4A, B). Treatment with the JNK inhibitor SP600125 inhibited rLCN2 activation of the JNK pathway (Fig. 4C-E). SP600125 also partially mitigated the rLCN2-induced reductions in SLC7A11 and GPX4 expression in 661 W cells (Fig. 4C,F,G). The MDA and GSH assays demonstrated that SP600125 alleviated the increase in MDA and the decrease in GSH levels induced by rLCN2 (Fig. 4H, I). In light-exposed 661 W cells, LCN2 knockdown by siLCN2 counteracted the light-induced JNK pathway activation (Fig. 4J-L). Collectively, these findings suggest that LCN2 triggers ferroptosis in 661 W cells through the JNK signaling pathway.

### LCN2 inhibition alleviated retinal ferroptosis and protected the retina from light-induced retinal degeneration

**Fig. 5:**
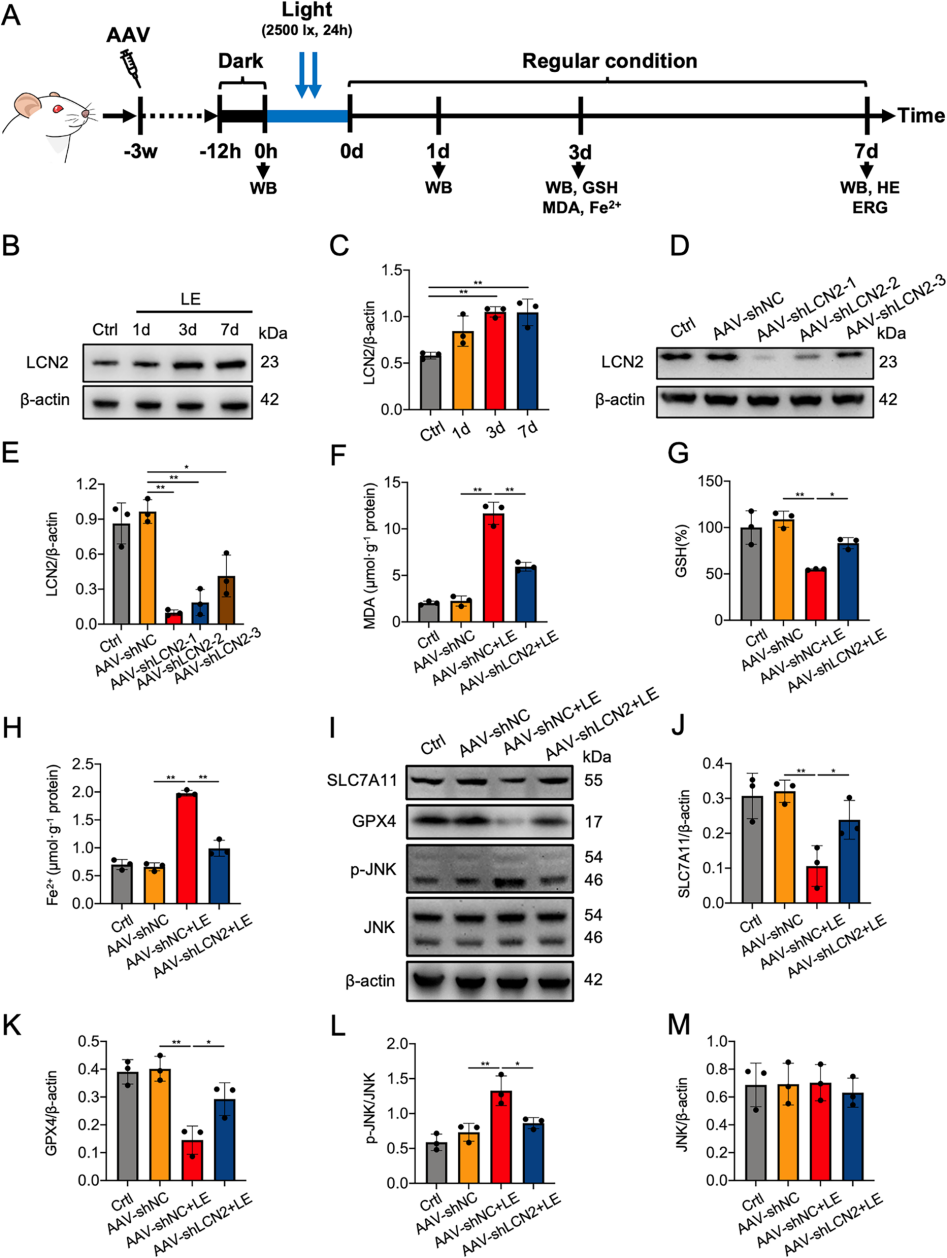
LCN2 knockdown alleviated ferroptosis and JNK pathway activation in the neural retina in vivo. **A** Timeline of the experimental design. **B**, **C** Western blotting and quantitative analysis of neural retinal LCN2 protein expression at 1, 3, and 7 days after light exposure (LE). The protein expression levels of LCN2 were normalized to those of β-actin and are presented as fold changes. **D**, **E** Western blotting and quantitative analysis of LCN2 protein expression in the neural retinas of rats following transduction with different AAV-shLCN2 sequences. The protein expression levels of LCN2 were normalized to those of β-actin and are presented as fold changes. **F** MDA levels in the indicated groups at 3 days after LE. **G** GSH levels in the indicated groups at 3 days after LE. **H** Intracellular Fe^2+^ levels in the indicated groups at 3 days after LE. **I**‒**M** Western blotting and quantitative analysis, showing that LCN2 knockdown inhibited the LE-induced increase in SLC7A11 and GPX4 and JNK phosphorylation at 3 days after LE. The protein expression levels of p-JNK were normalized to those of JNK and are presented as fold changes. The protein expression levels of JNK, SLC7A11, and GPX4 were normalized to those of β-actin and are presented as fold changes. *n* = 3 per group. *P < 0.05, **P < 0.01. One-way ANOVA followed by Tukey’s post hoc test

**Fig. 6:**
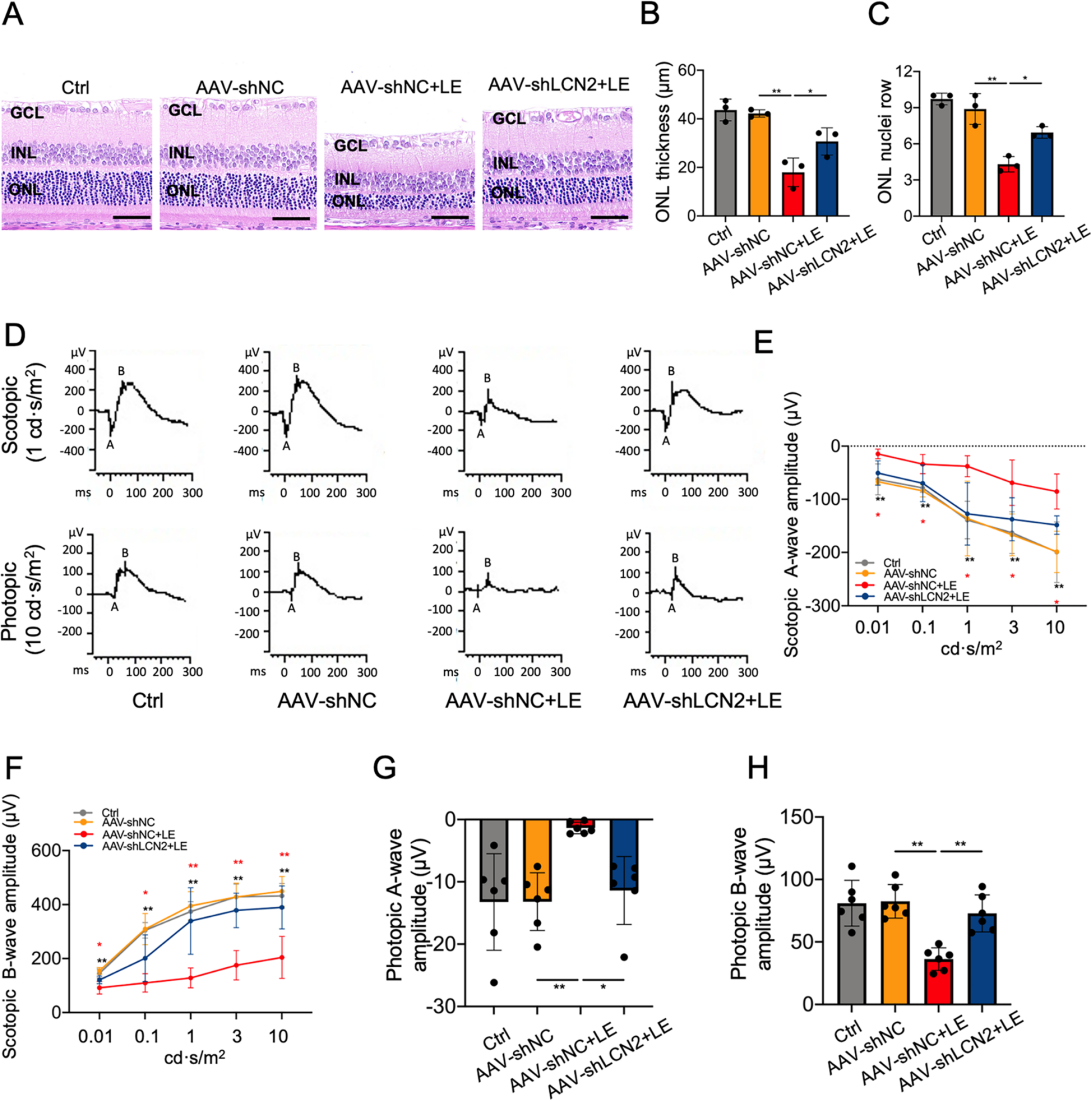
LCN2 knockdown protected the retinal structure and function in vivo. **A**-**C** Hematoxylin and eosin staining and quantitative analysis, showing that AAV-shLCN2 ameliorated the reduction in the thickness and the number of nuclei rows of ONL at 7 days after light exposure (LE). GCL: ganglion cell layer; INL: inner nuclear layer; ONL: outer nuclear layer. Scale bars = 50 μm. **D**‒**H** ERG was used to detect retinal function under scotopic and photopic conditions at 7 days after LE. Representative scotopic ERG at 1 cd·s/m^2^ and photopic ERG at 10 cd·s/m^2^ were shown (D). For scotopic ERG, rats were stimulated with flashes of ranging light intensity (0.01, 0.1, 1, 3 and 10 cd·s/m^2^). Under baseline conditions, no statistically significant differences were observed in scotopic a-wave or b-wave amplitudes between the control group and the AAV-shNC group across all tested stimulus intensities (0.01, 0.1, 1, 3, and 10 cd·s/m^2^; E and F). Following LE treatment, the AAV-shNC + LE group demonstrated marked attenuation of both a-wave and b-wave amplitudes compared to the AAV-shNC group at every intensity level (black **P < 0.01 vs. AAV-shNC). Notably, AAV-shLCN2 administration in the AAV-shLCN2 + LE group exhibited statistically significant recovery of a-wave and b-wave amplitudes compared to the AAV-shNC + LE group at every intensity level (red *P < 0.05, **P < 0.01 vs. AAV-shNC + LE). For photopic ERG, rats were stimulated with light intensity of 10 cd·s/m^2^. AAV-shLCN2 significantly suppressed the reductions in the amplitudes of the a and b waves at light intensity of 10 cd·s/m^2^ under photopic conditions (G and H). *n* = 6 per group. *P < 0.05, **P < 0.01. One-way ANOVA followed by Tukey’s post hoc test

In the light-induced retinal degeneration model (Fig. 5A), the neural retinal expression of LCN2 protein increased in a time-dependent manner, with significant increases at 3 and 7 days after light exposure (Fig. 5B,C). To determine the pro-ferroptotic function of LCN2 *in vivo*, we performed targeted LCN2 knockdown using AAV2/2-EGFP-delivered shRNA (AAV-shLCN2). Retinal cryosections demonstrated efficient transduction of AAV-shLCN2 in both photoreceptors and RPE cells at three weeks post subretinal injection, consistent with the known dual tropism of AAV2 for these cell types in rodents (Additional file 4). Because administration of AAV-shLCN2-1 reduced neural retinal LCN2 expression (Fig. 5D,E), we used it in the subsequent experiments. Western blotting analysis further demonstrated significant downregulation of LCN2 protein expression at 1, 3, and 7 days following light exposure in AAV-shLCN2-treated neural retinas, confirming the efficacy of LCN2 knockdown (Additional file 5). However, AAV-shLCN2 treatment did not alter the protein expression levels of GPX4 and SLC7A11 or the contents of MDA, GSH, or Fe^2+^ in neural retina under physiological conditions (Additional file 6). Three days post light exposure, AAV-shLCN2 treatment resulted in marked reduction of LCN2 immunofluorescence throughout the retina, with the most pronounced decrease observed in the outer retinal layers containing photoreceptors, compared with AAV-shNC treatment (Additional file 7). The MDA and GSH assays demonstrated that AAV-shLCN2 alleviated light-induced lipid peroxidation and GSH depletion at 3 days after light exposure (Fig. 5F,G). Furthermore, AAV-shLCN2 decreased retinal Fe^2+^ accumulation at 3 days after light exposure (Fig. 5H). Western blotting showed that AAV-shLCN2 upregulated GPX4 and SLC7A11 protein expression and attenuated JNK phosphorylation at 3 days after light exposure compared with neural retinas treated with AAV-shNC (Fig. 5I-M). We also examined the effects of LCN2 knockdown on retinal structure and function. Histological evaluation with H&E staining revealed that AAV-shLCN2 effectively mitigated photoreceptor atrophy and prevented the reduction in the thickness and the number of nuclei of the ONL in rats at 7 days after light exposure (Fig. 6A-C). Compared with AAV-shNC, administration of AAV-shLCN2 suppressed the reduction in the amplitudes of the ERG a and b waves at 7 days after light exposure (Fig. 6D-H). Furthermore, the decrease in the protein expression of SLC7A11 and GPX induced by light exposure in neural retinas and the reduction in the thickness and the number of nuclei row of the ONL were significantly inhibited by intravitreal administration of SP600125, indicating inhibition of JNK pathway was also protective for photoreceptors in vivo (Additional file 8). Collectively, our findings indicate that LCN2 knockdown alleviates retinal ferroptosis and protects the retina from light-induced degeneration, at least partly, through the JNK signaling pathway.

## Discussion

Photoreceptor loss is a common endpoint in various vision-threatening degenerative retinal diseases, including AMD and RP (Curcio et al. 1996; Hartong et al. 2006). There are currently no effective treatments to prevent or mitigate the loss of photoreceptors. In this study, we present direct in vitro and in vivo evidence demonstrating that LCN2 is a critical inducer of photoreceptor ferroptosis triggered by light exposure. We revealed that iron overload and activation of the JNK pathway are key components in the pathogenesis of LCN2-induced photoreceptor ferroptosis.

Multiple cell death pathways have been implicated in retinal cell death associated with retinal degeneration, including apoptosis (Wenzel et al. 2005), necroptosis (Murakami et al. 2014), and ferroptosis (Wenzel et al. 2005). Ferroptosis is a recently identified form of programmed cell death that depends on iron and is characterized by the accumulation of lipid peroxides (Dixon et al. 2012). Increasing evidence has highlighted its critical role in retinal cell injury and degeneration (Liu et al. 2023a). In vitro, ferroptosis in RPE cells and 661 W photoreceptor cells has been induced by various stimuli, such as H_2_O_2_ (Neiteler et al. 2023), interferon-γ (Wei et al. 2022), and atRAL (Yang et al. 2024a); these findings have been corroborated in animal models with dry AMD-like phenotypes, including sodium iodate-induced AMD and Abca4^−/−^ Rdh8^−/−^ mice (Tang et al. 2021b; Xiang et al. 2024). Our previous studies demonstrated that ferrostatin-1 can prevent photoreceptor ferroptosis in light-induced retinal degeneration (Tang et al. 2021a), although the underlying mechanisms are not fully understood.

An important finding of the present study is that light exposure significantly upregulated LCN2 expression, which in turn promoted photoreceptor ferroptosis. According to the literature, LCN2 production is typically low in normal conditions. However, in response to injury, infection, or other inflammatory stimuli, LCN2 expression is rapidly elevated (Abella et al. 2015). Notably, LCN2 has emerged as a molecular double-edged sword in inflammatory responses, exhibiting context-dependent functions. In acute inflammatory settings such as endotoxin-induced uveitis, LCN2 demonstrated protective anti-inflammatory properties by suppressing NF-κB p65 phosphorylation and nuclear translocation in Müller cells and retinal tissues (Tang et al. 2018a). Conversely, under chronic inflammatory conditions, age-associated LCN2 elevation appeared to promote pathological inflammation, as evidenced by increased CCL2 expression, reactive gliosis, and immune cell infiltration in the Cryba1 cKO mouse model of AMD-like pathology (Valapala et al. 2014). The roles of LCN2 in ferroptosis align with this dualistic paradigm. In a mouse intracerebral hemorrhage model, increased LCN2 inhibited the function of system Xc^−^, thereby promoting ferroptosis (Liu et al. 2023b). In a mouse model of dry AMD, an increase in LCN2 expression in RPE cells decreased autophagy and activated inflammasome‒ferroptosis processes (Gupta et al. 2023). Interestingly, although LCN2 induction has been linked to the promotion of ferroptosis, some studies indicate that LCN2 overexpression can mitigate ferroptotic cell death, suggesting that LCN2 plays distinct roles in different contexts (Chaudhary et al. 2021; Deng et al. 2024). In our study, LCN2 induced Fe^2+^ accumulation and lipid peroxidation, classical hallmarks of ferroptosis, in 661 W cells and neural retinal tissues. Conversely, LCN2 inhibition protected against these effects. Additionally, LCN2 knockdown in vivo alleviated photoreceptor loss and retinal dysfunction caused by light exposure. These findings indicate that LCN2 is a crucial proferroptotic mediator in light-induced photoreceptor degeneration.

Iron accumulation and oxidative damage are well-established pathogenic factors in AMD (Dunaief 2006). Notably, LCN2 can bind to iron, and the LCN2-iron complex can be internalized by binding to 24p3R and/or megalin, two potential LCN2 receptors (Devireddy et al. 2005; Hvidberg et al. 2005). This increased intracellular iron uptake is associated with elevated levels of ROS and exacerbation of lipid peroxidation via the Fenton reaction (Liu et al. 2022). Huang et al. demonstrated that in H9c2 cardiomyocytes, LCN2 administration induced ferroptosis by increasing the labile iron pool, whereas 24p3R knockdown significantly mitigated lipid peroxidation and reduced the expression of ferroptosis markers (Huang et al. 2022). In our study, we investigated the cellular and neural retinal iron levels, along with the associated oxidative stress markers, to assess whether LCN2 induced ferroptosis by regulating iron content. Cellular experiments demonstrated that the administration of rLCN2 significantly increased cellular Fe^2+^ uptake, ROS levels, and MDA levels in 661 W cells. Conversely, LCN2 knockdown using siRNA elicited the protected against these effects. In vivo studies using rats further indicated that LCN2 inhibition through shRNA reduced light-induced increases in neural retinal Fe^2+^ and MDA levels. These findings suggest that elevated levels of LCN2 may mediate ferroptosis by promoting iron overload, thereby enhancing cellular sensitivity to ferroptosis.

The members of the JNK family belong to the mitogen-activated protein kinase superfamily. The JNK signaling pathway is activated by various stimuli, including cytokines, growth factors, and stressors (Kim and Choi 2010). The pathway has been implicated in multiple biological functions, including cell proliferation and programmed cell death processes, such as apoptosis, autophagic cell death, and ferroptosis (Dhanasekaran and Reddy 2017). Yang et al. demonstrated that JNK inhibition significantly rescued photoreceptor cells from ferritinophagy-induced ferroptosis induced by atRAL (Yang et al. 2025). Additionally, pharmacological inhibition of JNK with SP600125 partially reversed the β-lapachone-induced decrease in SLC7A11 and GPX4 expression levels in colorectal cancer cells (Zhao et al. 2024). SLC7A11 is a light-chain subunit of system Xc^−^ (a cystine/glutamate antiporter system), which is essential for cystine uptake and cysteine synthesis, a rate-limiting precursor in intracellular GSH synthesis (Wu et al. 2004). GSH is a major antioxidant and affects GPX4 activity (Ursini and Maiorino 2020). The inhibition of SLC7A11 expression can impair the function of system Xc^−^ and inhibit extracellular cystine uptake, thereby hindering GSH synthesis. This reduces GPX4 activity and ultimately leads to the accumulation of lipid peroxides, resulting in ferroptosis (Koppula et al. 2021). In this study, we found that LCN2 activated the JNK signaling pathway and that knockdown of LCN2 in vivo inhibited the JNK pathway activation after light exposure. Importantly, SP600125 abolished LCN2-induced suppression of the SLC7A11-GSH-GPX4 axis and the subsequent increase in lipid peroxidation in 661 W cells. Together, these results suggest that LCN2-induced ferroptosis is mediated by activation of the JNK signaling pathway in photoreceptor cells.

This study has several limitations. First, a key limitation arises from the intensity disparity between in vivo and in vitro models. While 2500 lx light exposure in vivo induced photodamage, in vitro experiments demonstrated that this same intensity failed to elicit significant LCN2 expression in 661 W cells (Additional file 9). This differential response might result from reduced oxidative stress sensitivity in 661 W cells in vitro and a lack of synergistic stress amplification that occurs through multicellular interactions in retinal tissue. Nevertheless, intermediate light intensities (e.g., 3000–5000 lx) in vitro to effectively induce photoreceptor degeneration while maintaining better physiological relevance should be explored in future studies. Second, the exact mechanisms underlying the regulation of the SLC7A11-GSH-GPX4 axis by the JNK pathway has not been identified. Third, other modes of cell death, such as apoptosis and necrosis, have been identified as major contributors to retinal degeneration and their relationship to LCN2-induced ferroptosis should be investigated in further studies. Fourth, we utilized male rats exclusively in the current study. However, we acknowledge that future investigations should incorporate both sexes to comprehensively evaluate potential sex-specific effects of LCN2 in retinal ferroptosis. Fifth, although the 1 μg/mL rLCN2 concentration aligns with established in vitro neurotoxicity thresholds (Bi et al. 2013; Yoneshige et al. 2021), future studies should employ dose-escalation approaches to identify the minimal effective dose for ferroptosis induction. Such optimization could enhance clinical translatability while minimizing off-target cytotoxic effects. Moreover, while our study focused on photoreceptor-specific pathways, the potential contribution of RPE to light-induced retinal damage warrants further investigation. Finally, while light-induced retinal degeneration serves as an established model system (Grimm and Reme 2019), comprehensive evaluation of LCN2's role in retinal degeneration would benefit from additional experimental approaches, including pigmented animal models, genetic models recapitulating AMD or RP pathology and LCN2-knockout models.

## Conclusions

**Fig. 7:**
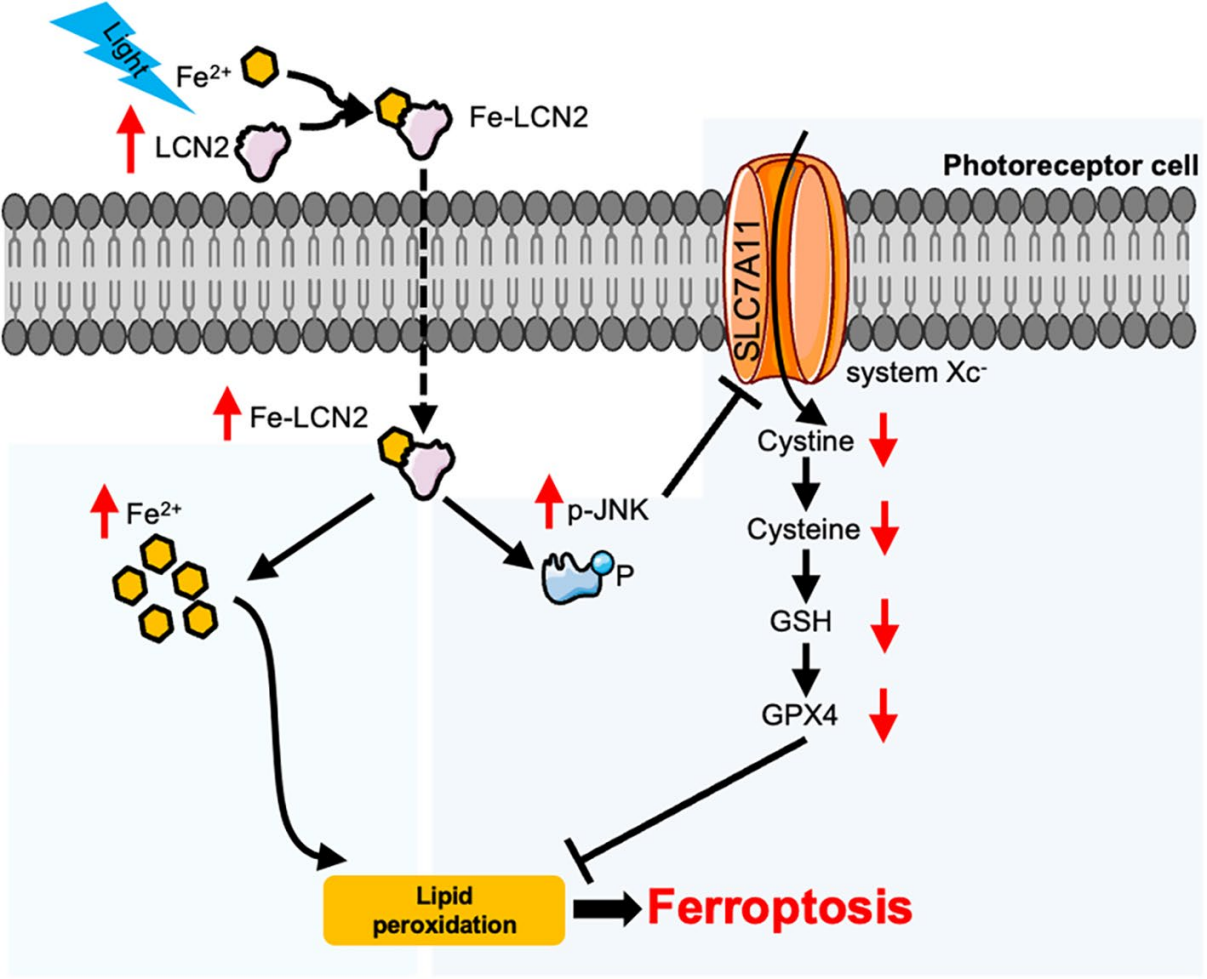
Proposed mechanism of LCN2 in the regulation of ferroptosis in photoreceptor cells. Light exposure induces LCN2 expression, which increases the intracellular Fe^2+^ level, promotes JNK phosphorylation, and subsequently inhibits the SLC7A11-GSH-GPX4 axis, resulting in ferroptosis in photoreceptor cells in retinal degeneration

Our results demonstrate that LCN2 induced ferroptosis in light-induced retinal degeneration by increasing the Fe^2+^ level and promoting the activation of JNK, and subsequent inhibition of the SLC7A11-GSH-GPX4 axis (Fig. 7). Our findings suggest that LCN2 may be a potential therapeutic target for retinal degeneration.

## Supplementary Information

Additional file 1.

Additional file 2.

Additional file 3.

Additional file 4.

Additional file 5.

Additional file 6.

Additional file 7.

Additional file 8.

Additional file 9.

## Data Availability

No datasets were generated or analysed during the current study.

## Abbreviations

AAV: Adreno-Associated Virus
atRAL: All-*trans*-retinal
AMD: Age-related macular degeneration
CCK-8: Cell counting kit-8
Ctrl: Control
DCFH-DA: 2′,7′-Dichlorodihydrofluorescein diacetate
DMEM: Dulbecco's modified Eagle's medium
DMSO: Dimethyl sulfoxide
ERG: Electroretinography
Fe^2+^: Ferrous
GCL: Ganglion cell layer
GPX4: Glutathione peroxidase 4
GSH: Glutathione
H&E: Hematoxylin–eosin
INL: Inner nuclear layer
JNK: C-Jun N-terminal kinase
LCN2: Lipocalin 2
LE: Light exposure
MDA: Malondialdehyde
NC: Negative control
ONL: Outer nuclear layer
PBS: Phosphate-buffered saline
rLCN2: Recombinant LCN2
ROS: Reactive oxygen species
RP: Retinitis pigmentosa
RPE: Retinal pigment epithelium
shRNA: Small hairpin ribonucleic acid
siRNA: Small interfering RNA
SLC7A11: Solute carrier family 7 member 11
TEM: Transmission electron microscopy

## References

[CR1] Abella V, et al. The potential of lipocalin-2/NGAL as biomarker for inflammatory and metabolic diseases. Biomarkers. 2015;20:565–71. 10.3109/1354750X.2015.1123354. 26671823 10.3109/1354750X.2015.1123354 PMC4819811

[CR2] Bi F, et al. Reactive astrocytes secrete lcn2 to promote neuron death. Proc Natl Acad Sci U S A. 2013;110:4069–74. 10.1073/pnas.1218497110. 23431168 10.1073/pnas.1218497110 PMC3593910

[CR3] Borkham-Kamphorst E, et al. Protective effects of lipocalin-2 (LCN2) in acute liver injury suggest a novel function in liver homeostasis. Biochim Biophys Acta. 2013;1832:660–73. 10.1016/j.bbadis.2013.01.014. 23376114 10.1016/j.bbadis.2013.01.014

[CR4] Brito M, et al. Understanding the Impact of Polyunsaturated Fatty Acids on Age-Related Macular Degeneration: A Review. Int J Mol Sci. 2024; 25. 10.3390/ijms25074099. 10.3390/ijms25074099 PMC11012607 38612907

[CR5] Chang GQ, Hao Y, Wong F. Apoptosis: final common pathway of photoreceptor death in rd, rds, and rhodopsin mutant mice. Neuron. 1993;11:595–605. 10.1016/0896-6273(93)90072-y. 8398150 10.1016/0896-6273(93)90072-y

[CR6] Chaudhary N, et al. Lipocalin 2 expression promotes tumor progression and therapy resistance by inhibiting ferroptosis in colorectal cancer. Int J Cancer. 2021;149:1495–511. 10.1002/ijc.33711. 34146401 10.1002/ijc.33711

[CR7] Chen C, et al. Ferroptosis drives photoreceptor degeneration in mice with defects in all-trans-retinal clearance. J Biol Chem. 2021;296:100187. 10.1074/jbc.RA120.015779. 33334878 10.1074/jbc.RA120.015779 PMC7948481

[CR8] Chen C, et al. Induction of ferroptosis by HO-1 contributes to retinal degeneration in mice with defective clearance of all-trans-retinal. Free Radic Biol Med. 2023;194:245–54. 10.1016/j.freeradbiomed.2022.12.008. 36509314 10.1016/j.freeradbiomed.2022.12.008

[CR9] Curcio CA, Medeiros NE, Millican CL. Photoreceptor loss in age-related macular degeneration. Invest Ophthalmol vis Sci. 1996;37:1236–49. 8641827

[CR10] Deng Y, et al. MafG/MYH9-LCN2 axis promotes liver fibrosis through inhibiting ferroptosis of hepatic stellate cells. Cell Death Differ. 2024;31:1127–39. 10.1038/s41418-024-01322-5. 38871948 10.1038/s41418-024-01322-5 PMC11369194

[CR11] Devireddy LR, Gazin C, Zhu X, Green MR. A cell-surface receptor for lipocalin 24p3 selectively mediates apoptosis and iron uptake. Cell. 2005;123:1293–305. 10.1016/j.cell.2005.10.027. 16377569 10.1016/j.cell.2005.10.027

[CR12] Dhanasekaran DN, Reddy EP. JNK-signaling: A multiplexing hub in programmed cell death. Genes Cancer. 2017; 8: 682–94. 10.18632/genesandcancer.155. 10.18632/genesandcancer.155 PMC5724802 29234486

[CR13] Dixon SJ, et al. Ferroptosis: an iron-dependent form of nonapoptotic cell death. Cell. 2012;149:1060–72. 10.1016/j.cell.2012.03.042. 22632970 10.1016/j.cell.2012.03.042 PMC3367386

[CR14] Dunaief JL. Iron induced oxidative damage as a potential factor in age-related macular degeneration: the Cogan Lecture. Invest Ophthalmol vis Sci. 2006;47:4660–4. 10.1167/iovs.06-0568. 17065470 10.1167/iovs.06-0568

[CR15] Flower DR. The lipocalin protein family: structure and function. Biochem J. 1996;318(Pt 1):1–14. 10.1042/bj3180001. 8761444 10.1042/bj3180001 PMC1217580

[CR16] Gao S, et al. Inhibition of Ferroptosis Ameliorates Photoreceptor Degeneration in Experimental Diabetic Mice. Int J Mol Sci. 2023; 24. 10.3390/ijms242316946. 10.3390/ijms242316946 PMC10707664 38069270

[CR17] Garcia-Baez J, et al. Developing a physiologically relevant cell model of ferroptosis in cardiomyocytes. Free Radic Biol Med. 2025;233:330–9. 10.1016/j.freeradbiomed.2025.04.006. 40185165 10.1016/j.freeradbiomed.2025.04.006

[CR18] Grimm C, Reme CE. Light Damage Models of Retinal Degeneration. Methods Mol Biol. 2019;1834:167–78. 10.1007/978-1-4939-8669-9_12. 30324444 10.1007/978-1-4939-8669-9_12

[CR19] Gu R, et al. Glucocorticoid-Induced Leucine Zipper Protects the Retina From Light-Induced Retinal Degeneration by Inducing Bcl-xL in Rats. Invest Ophthalmol vis Sci. 2017;58:3656–68. 10.1167/iovs.17-22116. 28728173 10.1167/iovs.17-22116

[CR20] Gupta U, et al. Increased LCN2 (lipocalin 2) in the RPE decreases autophagy and activates inflammasome-ferroptosis processes in a mouse model of dry AMD. Autophagy. 2023;19:92–111. 10.1080/15548627.2022.2062887. 35473441 10.1080/15548627.2022.2062887 PMC9809950

[CR21] Hartong DT, Berson EL, Dryja TP. Retinitis pigmentosa. Lancet. 2006;368:1795–809. 10.1016/S0140-6736(06)69740-7. 17113430 10.1016/S0140-6736(06)69740-7

[CR22] Huang Y, et al. Lipocalin-2 in neutrophils induces ferroptosis in septic cardiac dysfunction via increasing labile iron pool of cardiomyocytes. Front Cardiovasc Med. 2022;9:922534. 10.3389/fcvm.2022.922534. 35990970 10.3389/fcvm.2022.922534 PMC9386130

[CR23] Huang Z, et al. Tumor-secreted LCN2 impairs gastric cancer progression via autocrine inhibition of the 24p3R/JNK/c-Jun/SPARC axis. Cell Death Dis. 2024;15:756. 10.1038/s41419-024-07153-z. 39424639 10.1038/s41419-024-07153-z PMC11489581

[CR24] Hvidberg V, et al. The endocytic receptor megalin binds the iron transporting neutrophil-gelatinase-associated lipocalin with high affinity and mediates its cellular uptake. FEBS Lett. 2005;579:773–7. 10.1016/j.febslet.2004.12.031. 15670845 10.1016/j.febslet.2004.12.031

[CR25] Jiang X, Stockwell BR, Conrad M. Ferroptosis: mechanisms, biology and role in disease. Nat Rev Mol Cell Biol. 2021;22:266–82. 10.1038/s41580-020-00324-8. 33495651 10.1038/s41580-020-00324-8 PMC8142022

[CR26] Jiang C, et al. Targeting Lcn2 to Inhibit Myocardial Cell Ferroptosis is a Potential Therapy for Alleviating Septic Cardiomyopathy. Inflammation. 2025. 10.1007/s10753-025-02250-3. 39899131 10.1007/s10753-025-02250-3 PMC12596311

[CR27] Kim EK, Choi EJ. Pathological roles of MAPK signaling pathways in human diseases. Biochim Biophys Acta. 2010;1802:396–405. 10.1016/j.bbadis.2009.12.009. 20079433 10.1016/j.bbadis.2009.12.009

[CR28] Kong K, et al. Circular RNA expression profile and functional analysis of circUvrag in light-induced photoreceptor degeneration. Clin Exp Ophthalmol. 2024;52:558–75. 10.1111/ceo.14355. 38282307 10.1111/ceo.14355

[CR29] Koppula P, Zhuang L, Gan B. Cystine transporter SLC7A11/xCT in cancer: ferroptosis, nutrient dependency, and cancer therapy. Protein Cell. 2021;12:599–620. 10.1007/s13238-020-00789-5. 33000412 10.1007/s13238-020-00789-5 PMC8310547

[CR30] Lee EK, et al. Inhibition of the proliferation and invasion of hepatocellular carcinoma cells by lipocalin 2 through blockade of JNK and PI3K/Akt signaling. Int J Oncol. 2011;38:325–33. 10.3892/ijo.2010.854. 21132267 10.3892/ijo.2010.854

[CR31] Liu J, Kang R, Tang D. Signaling pathways and defense mechanisms of ferroptosis. FEBS J. 2022;289:7038–50. 10.1111/febs.16059. 34092035 10.1111/febs.16059

[CR32] Liu K, Li H, Wang F, Su Y. Ferroptosis: mechanisms and advances in ocular diseases. Mol Cell Biochem. 2023a;478:2081–95. 10.1007/s11010-022-04644-5. 36617346 10.1007/s11010-022-04644-5

[CR33] Liu X, et al. Dihydromyricetin attenuates intracerebral hemorrhage by reversing the effect of LCN2 via the system Xc^-^ pathway. Phytomedicine. 2023b;115:154756. 10.1016/j.phymed.2023.154756. 37130481 10.1016/j.phymed.2023.154756

[CR34] Liu Q, et al. Ferroptosis Contributes to Microvascular Dysfunction in Diabetic Retinopathy. Am J Pathol. 2024;194:1078–89. 10.1016/j.ajpath.2024.01.019. 38417697 10.1016/j.ajpath.2024.01.019

[CR35] Lv C, et al. PKD knockdown mitigates Ang II-induced cardiac hypertrophy and ferroptosis via the JNK/P53 signaling pathway. Cell Signal. 2024;113: 110974. 10.1016/j.cellsig.2023.110974. 37972803 10.1016/j.cellsig.2023.110974

[CR36] Magtanong L, Ko PJ, Dixon SJ. Emerging roles for lipids in non-apoptotic cell death. Cell Death Differ. 2016;23:1099–109. 10.1038/cdd.2016.25. 26967968 10.1038/cdd.2016.25 PMC5399169

[CR37] Marc RE, et al. Extreme retinal remodeling triggered by light damage: implications for age related macular degeneration. Mol vis. 2008;14:782–806. 18483561 PMC2375357

[CR38] Mei T, et al. Lipocalin 2 induces visual impairment by promoting ferroptosis in retinal ischemia-reperfusion injury. Ann Transl Med. 2023;11:3. 10.21037/atm-22-3298. 10.21037/atm-22-3298 PMC9906199 36760251

[CR39] Murakami Y, et al. Photoreceptor cell death and rescue in retinal detachment and degenerations. Prog Retin Eye Res. 2013;37:114–40. 10.1016/j.preteyeres.2013.08.001. 23994436 10.1016/j.preteyeres.2013.08.001 PMC3871865

[CR40] Murakami Y, et al. Programmed necrosis, not apoptosis, is a key mediator of cell loss and DAMP-mediated inflammation in dsRNA-induced retinal degeneration. Cell Death Differ. 2014;21:270–7. 10.1038/cdd.2013.109. 23954861 10.1038/cdd.2013.109 PMC3890945

[CR41] Nair DSR, Thomas BB. Stem Cell-based Treatment Strategies for Degenerative Diseases of the Retina. Curr Stem Cell Res Ther. 2022;17:214–25. 10.2174/1574888X16666210804112104. 34348629 10.2174/1574888X16666210804112104 PMC9129886

[CR42] Neiteler A, Palakkan AA, Gallagher KM, Ross JA. Oxidative stress and docosahexaenoic acid injury lead to increased necroptosis and ferroptosis in retinal pigment epithelium. Sci Rep. 2023;13:21143. 10.1038/s41598-023-47721-5. 38036571 10.1038/s41598-023-47721-5 PMC10689458

[CR43] Ni YQ, et al. Neuroprotective effects of naloxone against light-induced photoreceptor degeneration through inhibiting retinal microglial activation. Invest Ophthalmol vis Sci. 2008;49:2589–98. 10.1167/iovs.07-1173. 18515588 10.1167/iovs.07-1173

[CR44] Perche O, Doly M, Ranchon-Cole I. Caspase-dependent apoptosis in light-induced retinal degeneration. Invest Ophthalmol vis Sci. 2007;48:2753–9. 10.1167/iovs.06-1258. 17525209 10.1167/iovs.06-1258

[CR45] Reme CE, et al. Apoptotic cell death in retinal degenerations. Prog Retin Eye Res. 1998;17:443–64. 10.1016/s1350-9462(98)00009-3. 9777646 10.1016/s1350-9462(98)00009-3

[CR46] Tang W, et al. Lipocalin 2 Suppresses Ocular Inflammation by Inhibiting the Activation of NF-kappabeta Pathway in Endotoxin-Induced Uveitis. Cell Physiol Biochem. 2018a;46:375–88. 10.1159/000488472. 29590655 10.1159/000488472

[CR47] Tang W, et al. Light-Induced Lipocalin 2 Facilitates Cellular Apoptosis by Positively Regulating Reactive Oxygen Species/Bim Signaling in Retinal Degeneration. Invest Ophthalmol vis Sci. 2018b;59:6014–25. 10.1167/iovs.18-25213. 30574656 10.1167/iovs.18-25213

[CR48] Tang W, et al. Ferrostatin-1 attenuates ferroptosis and protects the retina against light-induced retinal degeneration. Biochem Biophys Res Commun. 2021a;548:27–34. 10.1016/j.bbrc.2021.02.055. 33631670 10.1016/j.bbrc.2021.02.055

[CR49] Tang Z, et al. HO-1-mediated ferroptosis as a target for protection against retinal pigment epithelium degeneration. Redox Biol. 2021b;43: 101971. 10.1016/j.redox.2021.101971. 33895485 10.1016/j.redox.2021.101971 PMC8099560

[CR50] Tsuruma K, et al. Metallothionein-III deficiency exacerbates light-induced retinal degeneration. Invest Ophthalmol vis Sci. 2012;53:7896–903. 10.1167/iovs.12-10165. 23132798 10.1167/iovs.12-10165

[CR51] Ursini F, Maiorino M. Lipid peroxidation and ferroptosis: The role of GSH and GPx4. Free Radic Biol Med. 2020;152:175–85. 10.1016/j.freeradbiomed.2020.02.027. 32165281 10.1016/j.freeradbiomed.2020.02.027

[CR52] Valapala M, et al. Increased Lipocalin-2 in the retinal pigment epithelium of Cryba1 cKO mice is associated with a chronic inflammatory response. Aging Cell. 2014;13:1091–4. 10.1111/acel.12274. 25257511 10.1111/acel.12274 PMC4244249

[CR53] Varga D, Hajdinak P, Makk-Merczel K, Szarka A. The Possible Connection of Two Dual Function Processes: The Relationship of Ferroptosis and the JNK Pathway. Int J Mol Sci. 2022; 23. 10.3390/ijms231911004. 10.3390/ijms231911004 PMC9570426 36232313

[CR54] Wang X, et al. Deferoxamine attenuates visual impairment in retinal ischemia-reperfusion via inhibiting ferroptosis. Sci Rep. 2023;13:20145. 10.1038/s41598-023-46104-0. 37978208 10.1038/s41598-023-46104-0 PMC10656451

[CR55] Wang H, et al. STZ-induced diabetes exacerbates neurons ferroptosis after ischemic stroke by upregulating LCN2 in neutrophils. Exp Neurol. 2024;377:114797. 10.1016/j.expneurol.2024.114797. 38670252 10.1016/j.expneurol.2024.114797

[CR56] Wei TT, et al. Interferon-gamma induces retinal pigment epithelial cell Ferroptosis by a JAK1-2/STAT1/SLC7A11 signaling pathway in Age-related Macular Degeneration. FEBS J. 2022;289:1968–83. 10.1111/febs.16272. 34741776 10.1111/febs.16272

[CR57] Wenzel A, Grimm C, Samardzija M, Reme CE. Molecular mechanisms of light-induced photoreceptor apoptosis and neuroprotection for retinal degeneration. Prog Retin Eye Res. 2005;24:275–306. 10.1016/j.preteyeres.2004.08.002. 15610977 10.1016/j.preteyeres.2004.08.002

[CR58] Wu G, et al. Glutathione metabolism and its implications for health. J Nutr. 2004;134:489–92. 10.1093/jn/134.3.489. 14988435 10.1093/jn/134.3.489

[CR59] Wu Y, et al. Resveratrol protects retinal ganglion cell axons through regulation of the SIRT1-JNK pathway. Exp Eye Res. 2020;200:108249. 10.1016/j.exer.2020.108249. 32956685 10.1016/j.exer.2020.108249

[CR60] Xiang W, et al. PEDF protects retinal pigment epithelium from ferroptosis and ameliorates dry AMD-like pathology in a murine model. Geroscience. 2024;46:2697–714. 10.1007/s11357-023-01038-3. 38153666 10.1007/s11357-023-01038-3 PMC10828283

[CR61] Xiao X, Yeoh BS, Vijay-Kumar M. Lipocalin 2: An Emerging Player in Iron Homeostasis and Inflammation. Annu Rev Nutr. 2017;37:103–30. 10.1146/annurev-nutr-071816-064559. 28628361 10.1146/annurev-nutr-071816-064559

[CR62] Xiong M, et al. Qi-Shen-Tang alleviates retinitis pigmentosa by inhibiting ferroptotic features via the NRF2/GPX4 signaling pathway. Heliyon. 2023;9:e22443. 10.1016/j.heliyon.2023.e22443. 38034716 10.1016/j.heliyon.2023.e22443 PMC10687062

[CR63] Xu J, et al. Pregabalin Mediates Retinal Ganglion Cell Survival From Retinal Ischemia/Reperfusion Injury Via the Akt/GSK3beta/beta-Catenin Signaling Pathway. Invest Ophthalmol vis Sci. 2022;63:7. 10.1167/iovs.63.12.7. 36326725 10.1167/iovs.63.12.7 PMC9645359

[CR64] Yang B, et al. Inhibition of JNK signaling attenuates photoreceptor ferroptosis caused by all-trans-retinal. Free Radic Biol Med. 2025;227:179–89. 10.1016/j.freeradbiomed.2024.12.007. 39643129 10.1016/j.freeradbiomed.2024.12.007

[CR65] Yang B, Yang K, Chen J, Wu Y. Crocin Protects the 661W Murine Photoreceptor Cell Line against the Toxic Effects of All-Trans-Retinal. Int J Mol Sci. 2024a; 25. 10.3390/ijms251810124. 10.3390/ijms251810124 PMC11432120 39337609

[CR66] Ye Z, et al. Deferiprone protects photoreceptors by inhibiting ferroptosis after experimental retinal detachment. Exp Eye Res. 2025;250:110156. 10.1016/j.exer.2024.110156. 39549870 10.1016/j.exer.2024.110156

[CR67] Yoneshige A, et al. Elevated Hydrostatic Pressure Causes Retinal Degeneration Through Upregulating Lipocalin-2. Front Cell Dev Biol. 2021;9:664327. 10.3389/fcell.2021.664327. 34136483 10.3389/fcell.2021.664327 PMC8201777

[CR68] Yoshizawa K, et al. Caspase-3 inhibitor transiently delays inherited retinal degeneration in C3H mice carrying the rd gene. Graefes Arch Clin Exp Ophthalmol. 2002;240:214–9. 10.1007/s00417-002-0427-5. 11935279 10.1007/s00417-002-0427-5

[CR69] Zhang M, et al. Role of fractalkine/CX3CR1 interaction in light-induced photoreceptor degeneration through regulating retinal microglial activation and migration. PLoS ONE. 2012;7:e35446. 10.1371/journal.pone.0035446. 22536384 10.1371/journal.pone.0035446 PMC3334900

[CR70] Zhang Z, et al. Activation of ferritinophagy is required for the RNA-binding protein ELAVL1/HuR to regulate ferroptosis in hepatic stellate cells. Autophagy. 2018;14:2083–103. 10.1080/15548627.2018.1503146. 30081711 10.1080/15548627.2018.1503146 PMC6984765

[CR71] Zhang Z, et al. Retinal light damage: From mechanisms to protective strategies. Surv Ophthalmol. 2024;69:905–15. 10.1016/j.survophthal.2024.07.004. 39053594 10.1016/j.survophthal.2024.07.004

[CR72] Zhao L, et al. beta-Lapachone induces ferroptosis of colorectal cancer cells via NCOA4-mediated ferritinophagy by activating JNK pathway. Chem Biol Interact. 2024;389:110866. 10.1016/j.cbi.2024.110866. 38218311 10.1016/j.cbi.2024.110866

