# Supplementary material for: Lipocalin-2-mediated ferroptosis as a target for protection against light-induced photoreceptor degeneration
Source: Mol Med. 2025 May 15;31:190. doi: 10.1186/s10020-025-01250-1 (PMC12083120; doi:10.1186/s10020-025-01250-1)
Supplement: Supplementary file 1 — Additional file 1. [file 10020_2025_1250_MOESM1_ESM.pdf]

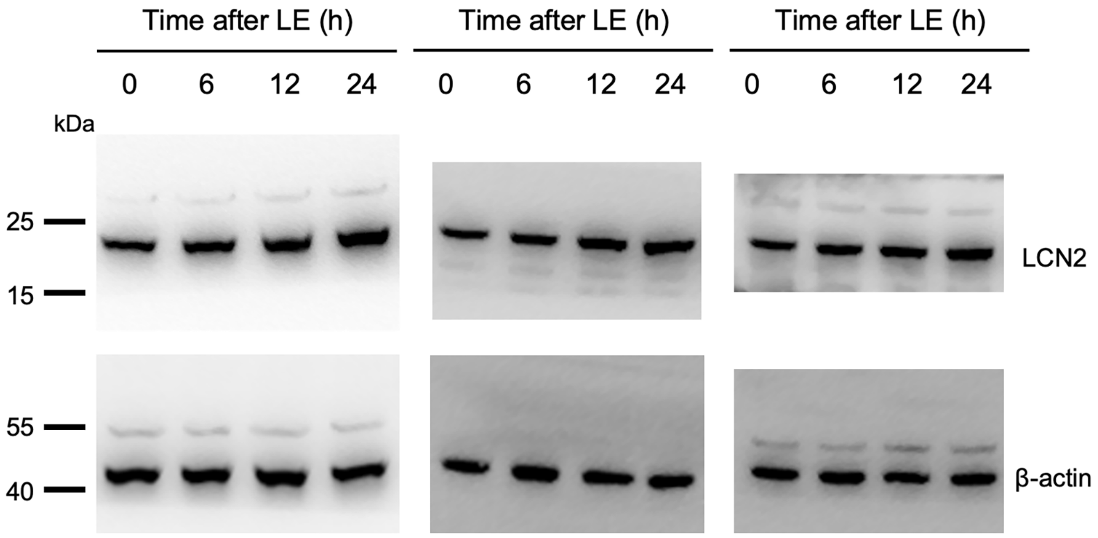


Western blot raw data for Figure 1B

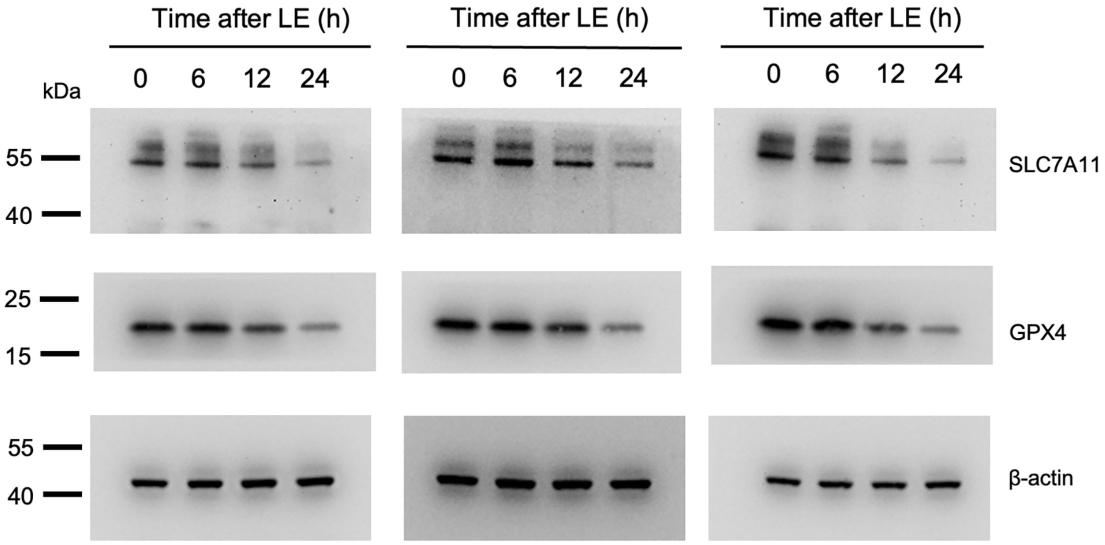


Western blot raw data for Figure 1D

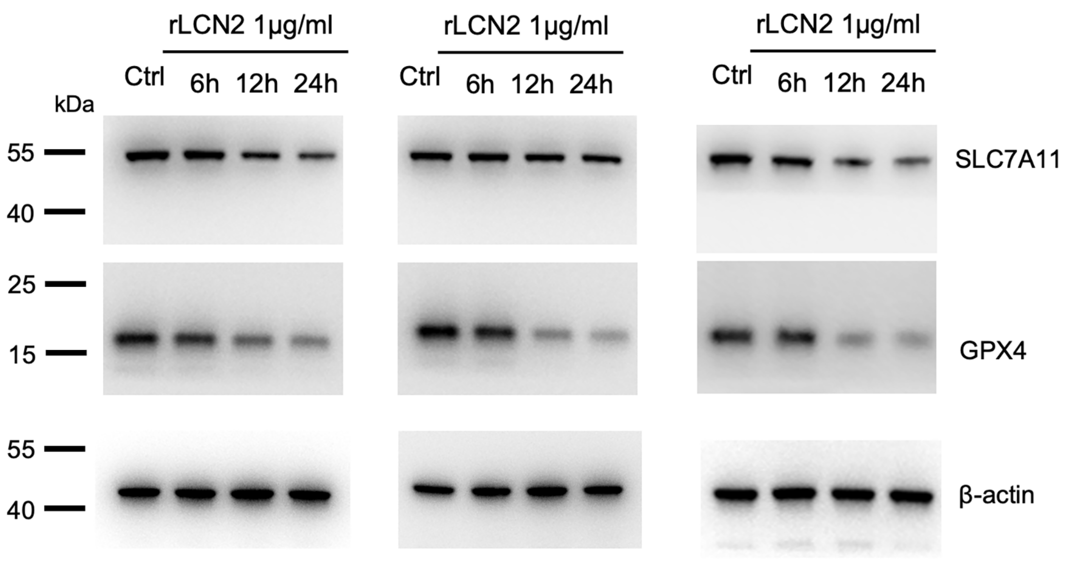


Western blot raw data for Figure 2B

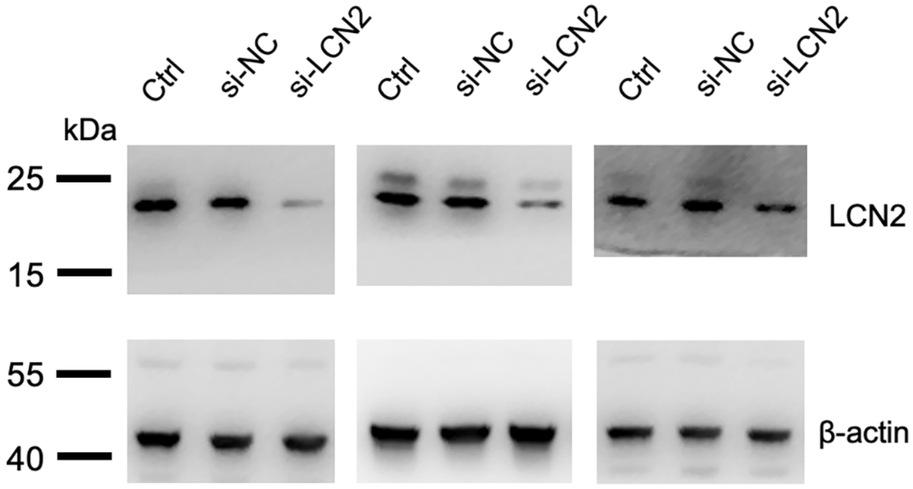


Western blot raw data for Figure 3A

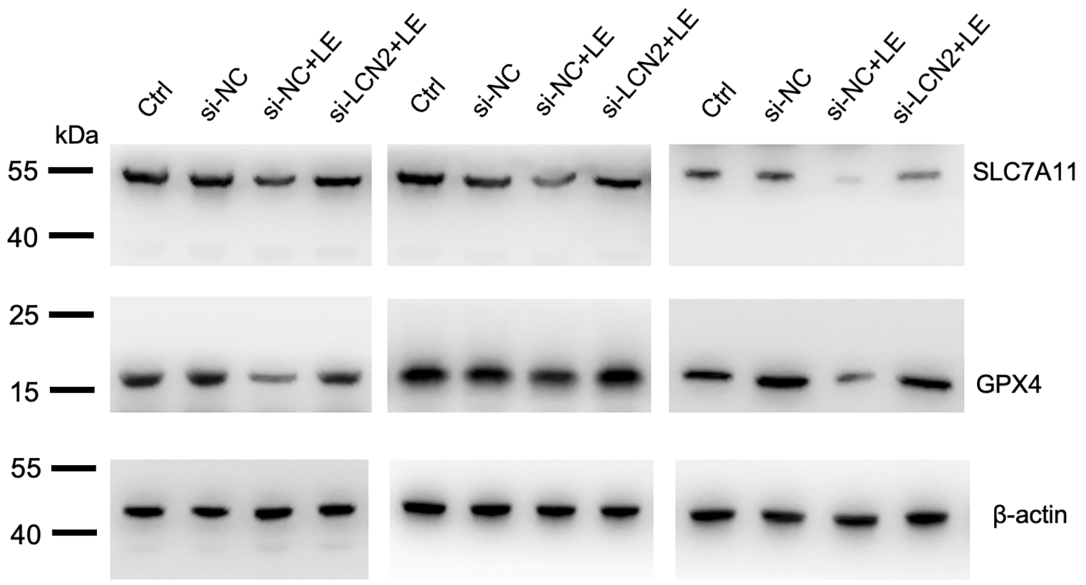


Western blot raw data for Figure 3I

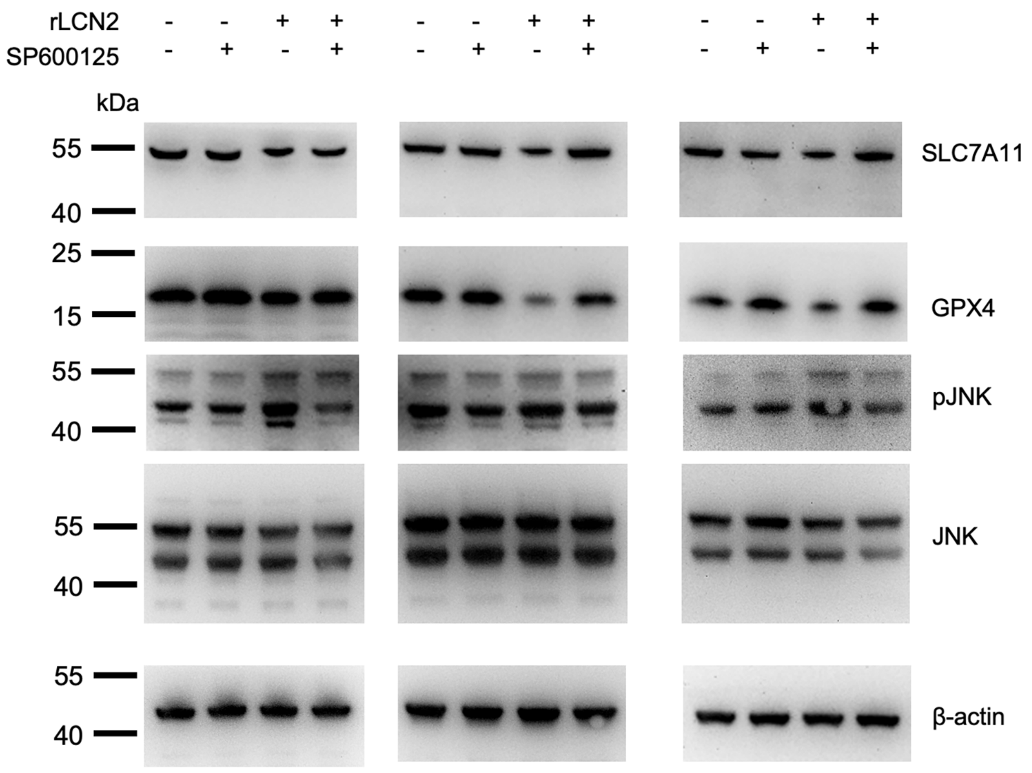


Western blot raw data for Figure 4C

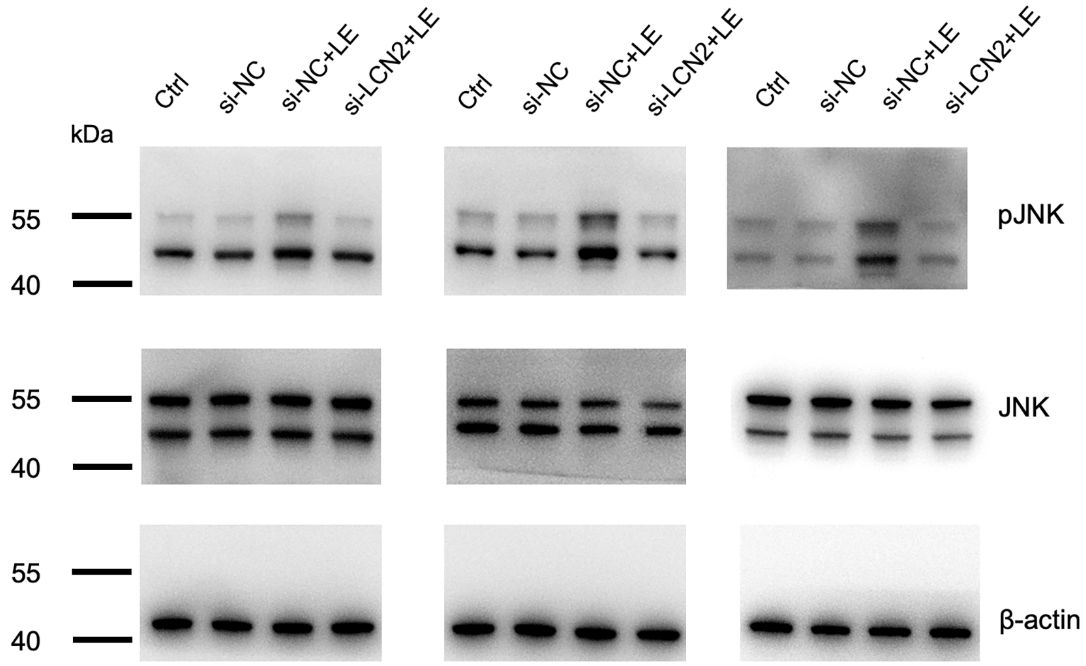


Western blot raw data for Figure 4J

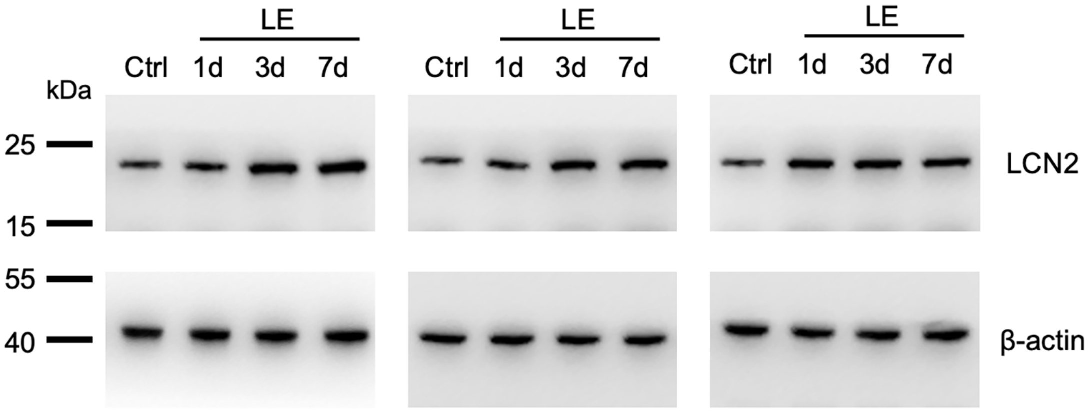


Western blot raw data for Figure 5B

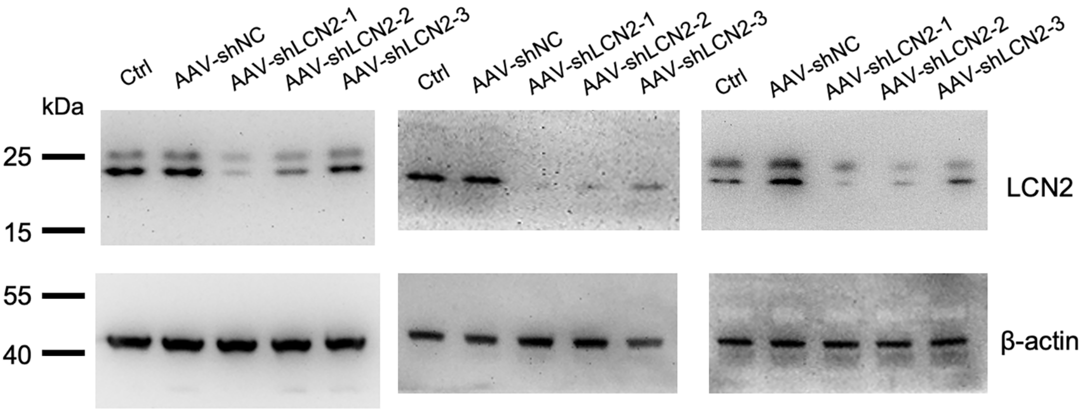


Western blot raw data for Figure 5D

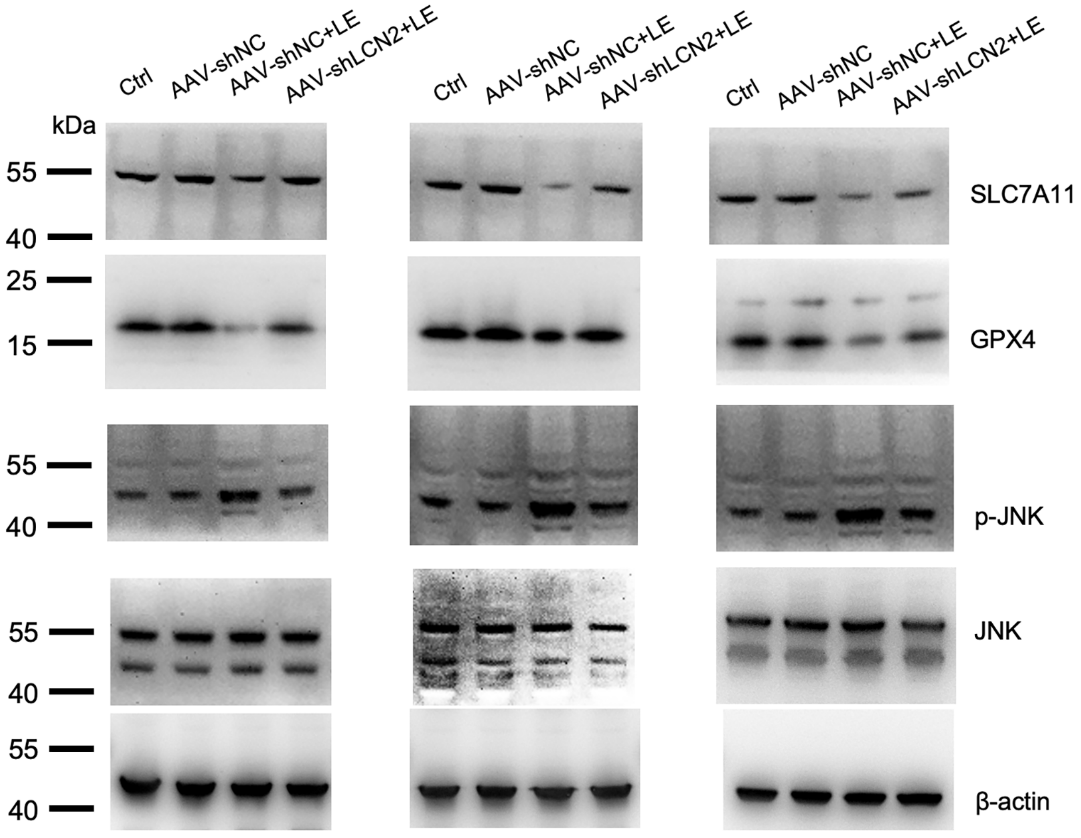


Western blot raw data for Figure 5I

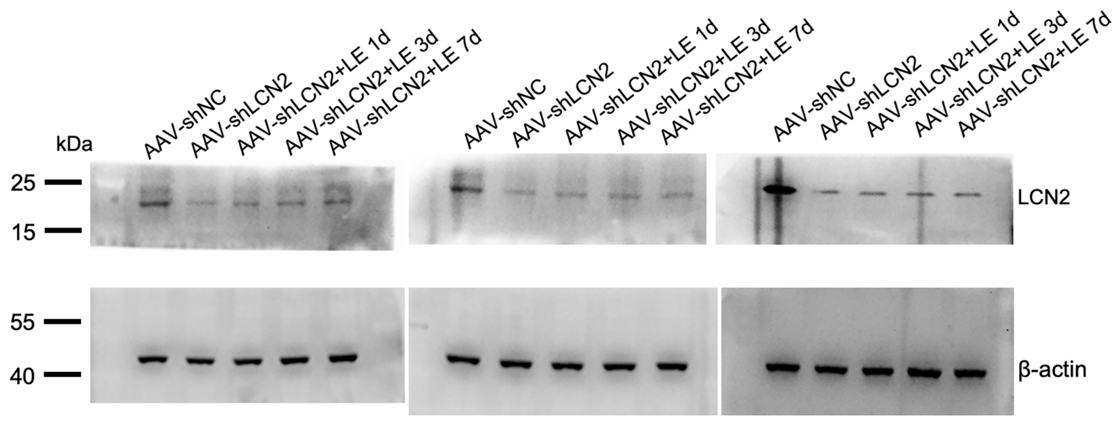


Western blot raw data for Additional file 5A

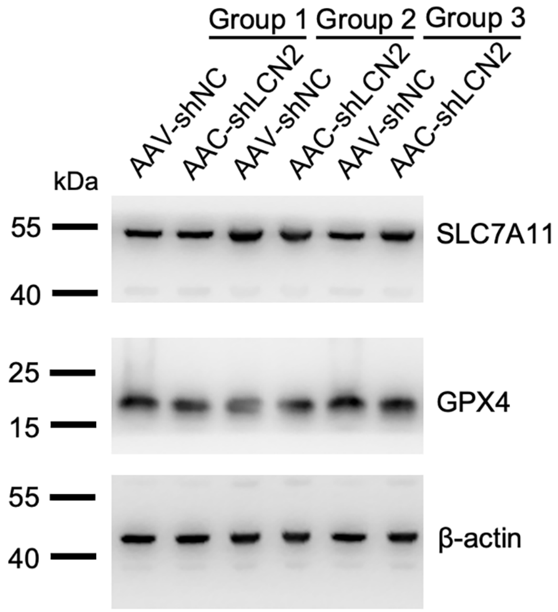


Western blot raw data for Additional file 6A

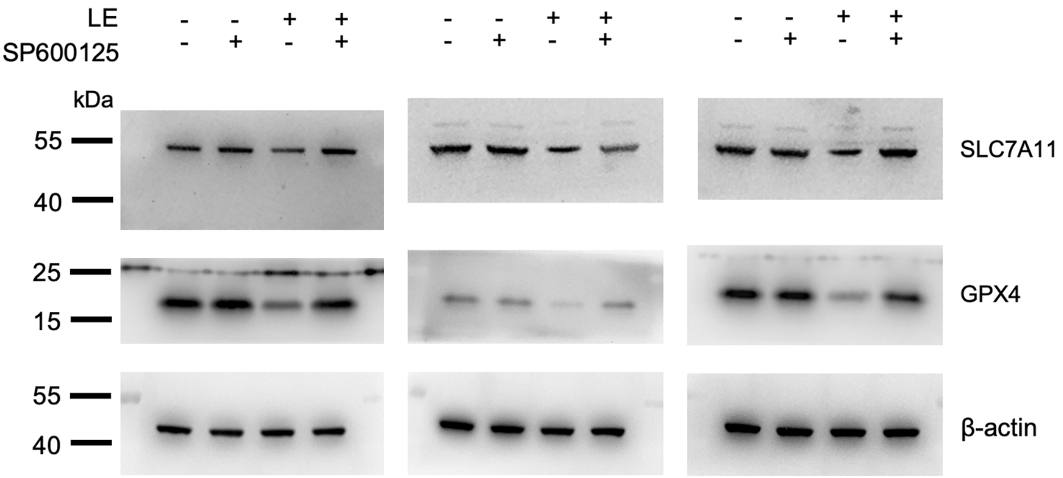


Western blot raw data for Additional file 8A

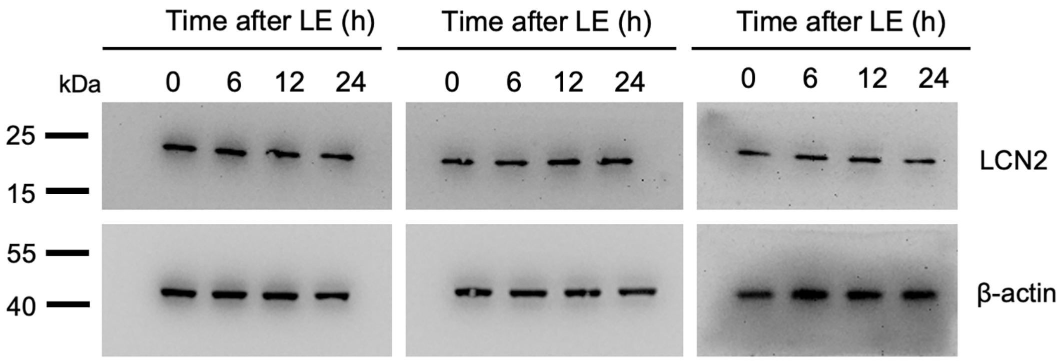


Western blot raw data for Additional file 9
