# Supplementary material for: Lipocalin-2-mediated ferroptosis as a target for protection against light-induced photoreceptor degeneration
Source: Mol Med. 2025 May 15;31:190. doi: 10.1186/s10020-025-01250-1 (PMC12083120; doi:10.1186/s10020-025-01250-1)
Supplement: Supplementary file 2 — Additional file 2. [file 10020_2025_1250_MOESM2_ESM.pdf]

Additional file 2: Numbers of rats utilized in study.

Step 1	Step 2	Number of Rats
None	None	12
	Light exposure	12
Subretinal injection of AAV-shNC	None	12
Subretinal injection of AAV-shLCN2-1	None	3
Subretinal injection of AAV-shLCN2-2	None	3
Subretinal injection of AAV-shLCN2-3	None	3
Subretinal injection of AAV-shLCN2*	None	3
Subretinal injection of AAV-shNC	Light exposure	6
Subretinal injection of AAV-shLCN2*	Light exposure	15
Intravitreal injection of SP600125	None	3
Intravitreal injection of SP600125	Light exposure	3
In total		75

*Because administration of AAV-shLCN2-1 reduced neural retinal LCN2 expression most remarkably, we used it in the subsequent experiments.
