# Supplementary material for: Lipocalin-2-mediated ferroptosis as a target for protection against light-induced photoreceptor degeneration
Source: Mol Med. 2025 May 15;31:190. doi: 10.1186/s10020-025-01250-1 (PMC12083120; doi:10.1186/s10020-025-01250-1)
Supplement: Supplementary file 3 — Additional file 3. [file 10020_2025_1250_MOESM3_ESM.pdf]

Additional file 3. The shRNA sequences targeting LCN2 and negative control containing scrambled short hairpin RNA (AAV-shNC) sequences

		Sequences
shRNA1	Top strand	AATTCGAATGCGGTCCAGAAAGAAAGACAACTCGAGTTGTCTTTCTTTCTGGACCGCATTCTTTTTTG
	Bottom strand	GATCCAAAAAAGAATGCGGTCCAGAAAGAAAGACAACTCGAGTTGTCTTTCTTTCTGGACCGCATTCG
shRNA2	Top strand	AATTCGCAGGAAGACAATAGCTACAACGTCACTCGAGTGACGTTGTAGCTATTGTCTTCCTGTTTTTTG
	Bottom strand	GATCCAAAAAACAGGAAGACAATAGCTACAACGTCACTCGAGTGACGTTGTAGCTATTGTCTTCCTGCG
shRNA3	Top strand	AATTCGCAGTACTTCAAAGTCACCCTGTACGCTCGAGCGTACAGGGTGACTTTGAAGTACTGTTTTTTG
	Bottom strand	GATCCAAAAAACAGTACTTCAAAGTCACCCTGTACGCTCGAGCGTACAGGGTGACTTTGAAGTACTGCG
NC shRNA	Top strand	GATCCGTTCTCCGAACGTGTCACGTAATTCAAGAGATTACGTGACACGTTCGGAGAATTTTTTC
	Bottom strand	AATTGAAAAAATTCTCCGAACGTGTCACGTAATCTCTTGAATTACGTGACACGTTCGGAGAACG
