# Supplementary material for: Lipocalin-2-mediated ferroptosis as a target for protection against light-induced photoreceptor degeneration
Source: Mol Med. 2025 May 15;31:190. doi: 10.1186/s10020-025-01250-1 (PMC12083120; doi:10.1186/s10020-025-01250-1)
Supplement: Supplementary file 4 — Additional file 4. [file 10020_2025_1250_MOESM4_ESM.pdf]

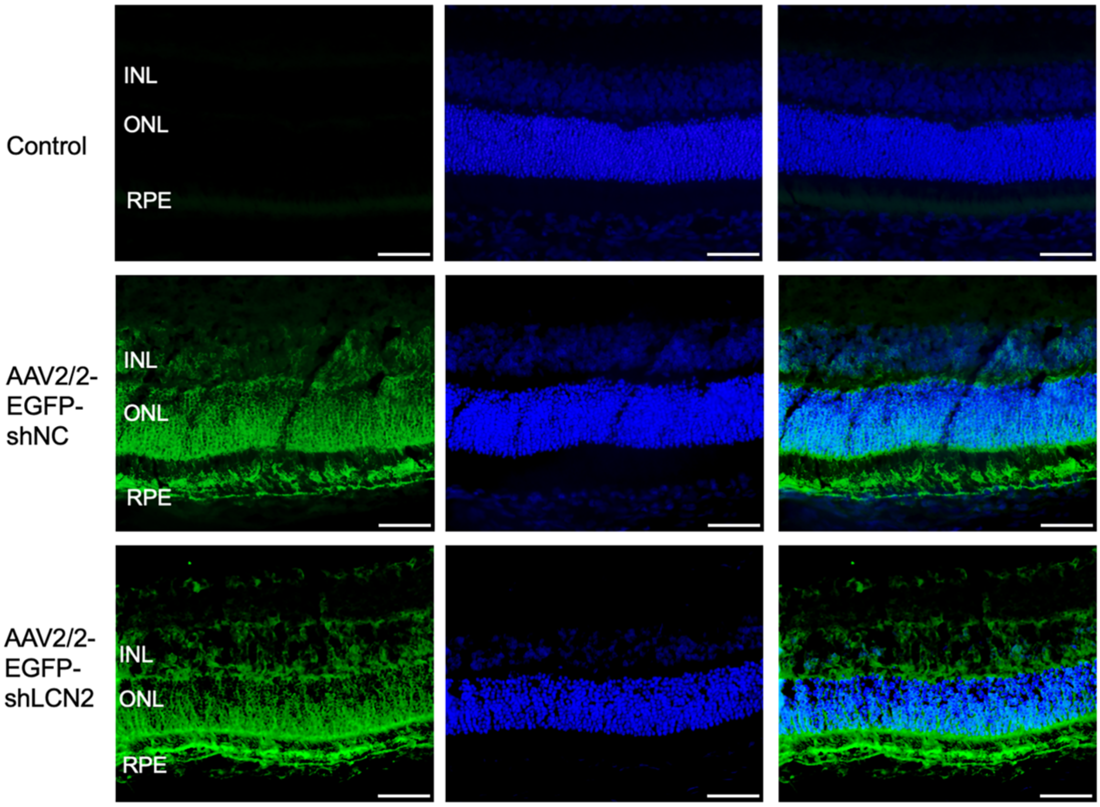

Additional file 4. Immunofluorescence analysis of retinal sections three weeks following subretinal injection demonstrated that both AAV2/2-EGFP-shNC and AAV2/2-EGFP-shLCN2 (green) predominantly transduced photoreceptor and retinal pigment epithelial (RPE) cells. Blue: DAPI; INL: inner nuclear layer; ONL: outer nuclear layer; RPE: retinal pigment epithelium. Scale bars: 50 μm.
