# Supplementary material for: Lipocalin-2-mediated ferroptosis as a target for protection against light-induced photoreceptor degeneration
Source: Mol Med. 2025 May 15;31:190. doi: 10.1186/s10020-025-01250-1 (PMC12083120; doi:10.1186/s10020-025-01250-1)
Supplement: Supplementary file 5 — Additional file 5. [file 10020_2025_1250_MOESM5_ESM.pdf]

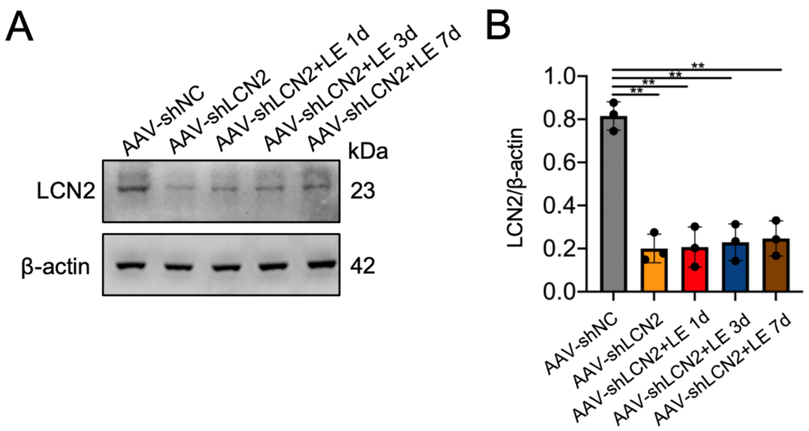

Additional file 5: LCN2 knockdown by AAV-shLCN2 after light exposure in the neural retina *in vivo*. A, B. Western blotting and quantitative analysis of LCN2 protein expression in the neural retinas of rats at 1, 3 and 7 days following light exposure (LE). The protein expression levels of LCN2 were normalized to those of β-actin and are presented as fold changes. $n = 3$ per group. $^{**}P < 0.01$. One-way ANOVA followed by Tukey's *post hoc* test.
