# Supplementary material for: Lipocalin-2-mediated ferroptosis as a target for protection against light-induced photoreceptor degeneration
Source: Mol Med. 2025 May 15;31:190. doi: 10.1186/s10020-025-01250-1 (PMC12083120; doi:10.1186/s10020-025-01250-1)
Supplement: Supplementary file 6 — Additional file 6. [file 10020_2025_1250_MOESM6_ESM.pdf]

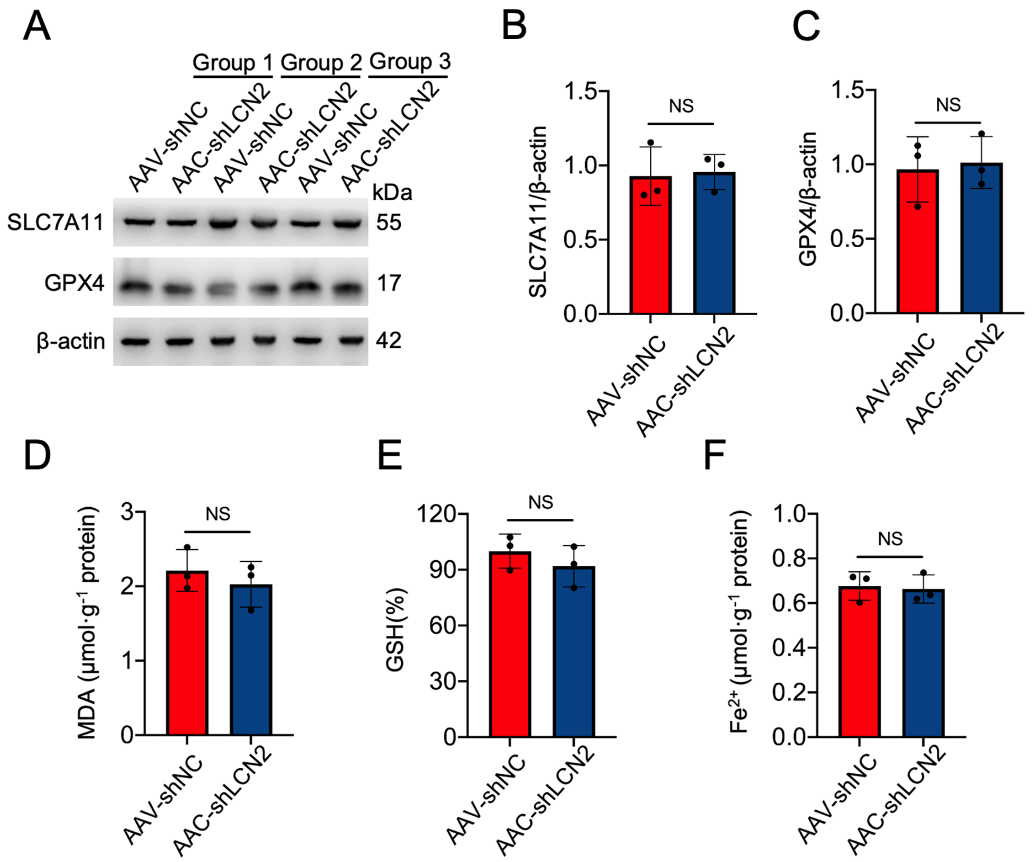

Additional file 6. LCN2 inhibition by AAV-shLCN2 treatment did not alter neural retinal protein expression of SLC7A11 and GPX4 (A-C) or MDA level (D) or GSH level (E) or Fe^{2+} content (F) without light exposure stimuli. The protein expression levels of SLC7A11 and GPX4 were normalized to those of β-actin and are presented as fold changes. *n* = 3 per group. Student's *t*-test. NS, not significant.
