# Supplementary material for: Lipocalin-2-mediated ferroptosis as a target for protection against light-induced photoreceptor degeneration
Source: Mol Med. 2025 May 15;31:190. doi: 10.1186/s10020-025-01250-1 (PMC12083120; doi:10.1186/s10020-025-01250-1)
Supplement: Supplementary file 7 — Additional file 7. [file 10020_2025_1250_MOESM7_ESM.pdf]

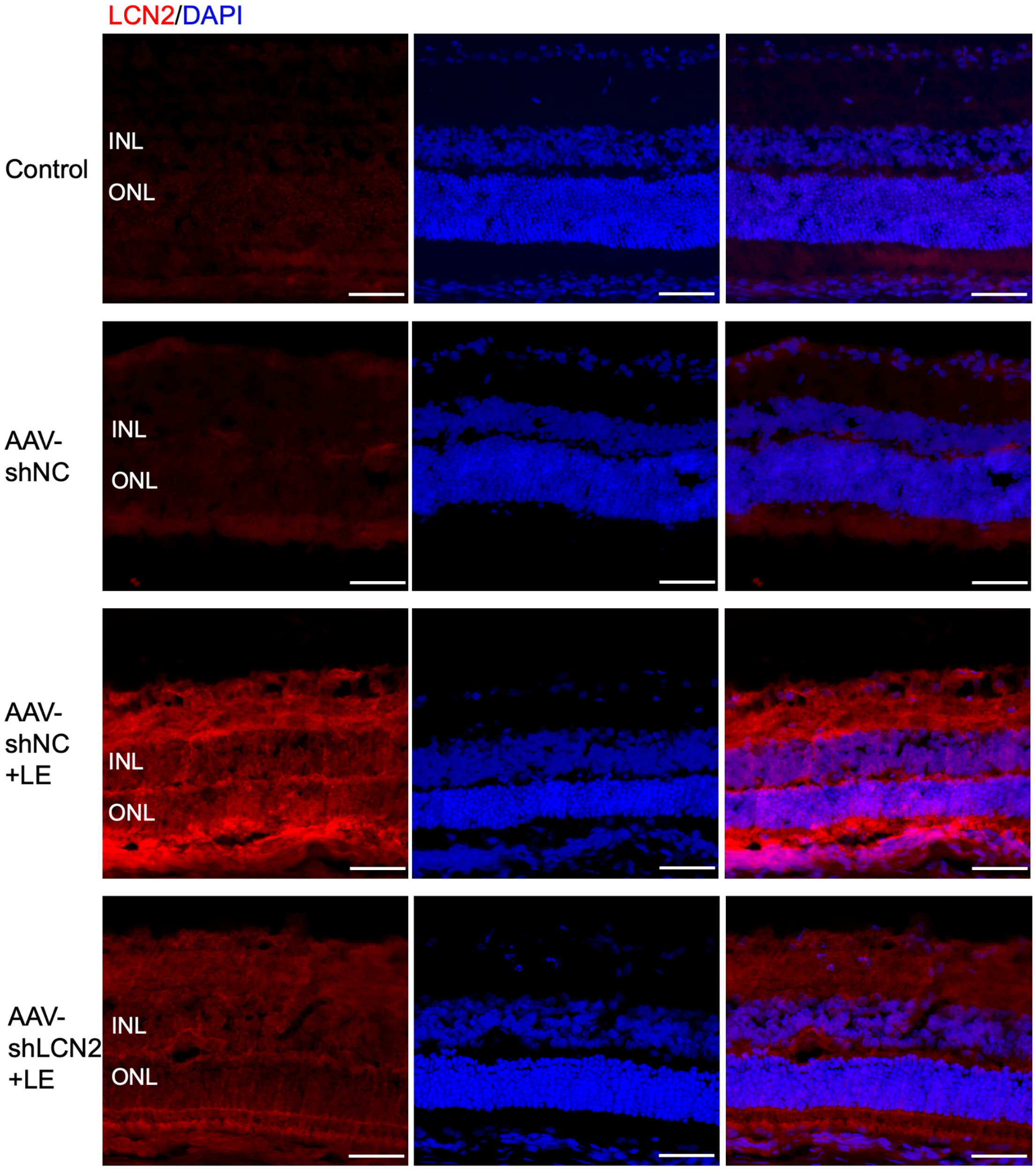

Additional file 7. Representative retinal sections immunostained with anti-LCN2 antibody (red) in different groups. AAV2-shLCN2 treatment reduced LCN2 immunoreactivity throughout retinal layers, with the most prominent reduction observed in the outer retina at 3 days after light exposure (LE). Blue: DAPI; INL: inner nuclear layer; ONL: outer nuclear layer. Scale bars: 50 μm.
