# Supplementary material for: Lipocalin-2-mediated ferroptosis as a target for protection against light-induced photoreceptor degeneration
Source: Mol Med. 2025 May 15;31:190. doi: 10.1186/s10020-025-01250-1 (PMC12083120; doi:10.1186/s10020-025-01250-1)
Supplement: Supplementary file 8 — Additional file 8. [file 10020_2025_1250_MOESM8_ESM.pdf]

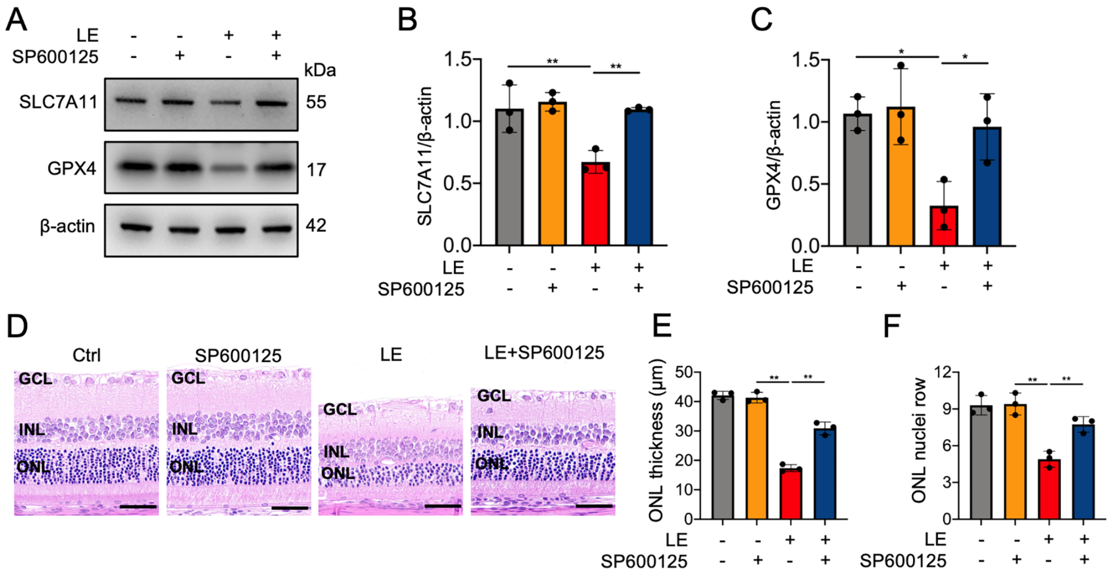

Additional file 8. SP600125 treatment provided neuroprotection for photoreceptors *in vivo*. A–C. SP600125 treatment significantly attenuated light-induced downregulation of SLC7A11 and GPX4 protein expression in neural retina at 3 days after light exposure (LE). The protein expression levels of SLC7A11 and GPX4 were normalized to those of β-actin and are presented as fold changes. D–F. SP600125 treatment prevented the reduction in outer nuclear layer (ONL) thickness and photoreceptor nuclei row count at 7 days after LE. GCL: ganglion cell layer; INL: inner nuclear layer; ONL: outer nuclear layer. Scale bars = 50 μm. $n = 3$ per group. *$P < 0.05$, **$P < 0.01$. One-way ANOVA followed by Tukey's *post hoc* test.
