# Supplementary material for: Lipocalin-2-mediated ferroptosis as a target for protection against light-induced photoreceptor degeneration
Source: Mol Med. 2025 May 15;31:190. doi: 10.1186/s10020-025-01250-1 (PMC12083120; doi:10.1186/s10020-025-01250-1)
Supplement: Supplementary file 9 — Additional file 9. [file 10020_2025_1250_MOESM9_ESM.pdf]

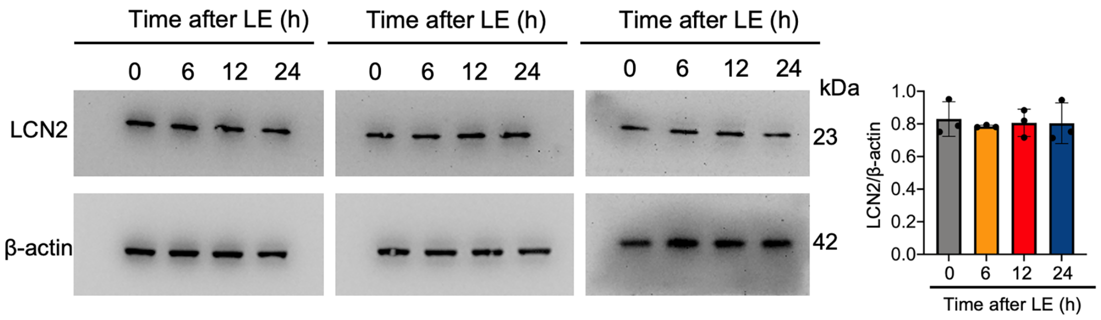

Additional file 9: Western blotting and quantitative analysis of LCN2 protein expression at 6, 12, and 24 h after light exposure (2500 lux, 5 h) in 661W cells. The protein expression levels of LCN2 were normalized to those of β-actin and are presented as fold changes. LE: light exposure. *n* = 3 per group. One-way ANOVA followed by Tukey's *post hoc* test.
